# Chemotherapy Drives Tertiary Lymphoid Structures That Correlate with ICI-Responsive TCF1^+^CD8^+^ T Cells in Metastatic Ovarian Cancer

**DOI:** 10.1158/1078-0432.CCR-24-1594

**Published:** 2024-08-20

**Authors:** Tereza Lanickova, Michal Hensler, Lenka Kasikova, Sarka Vosahlikova, Artemis Angelidou, Josef Pasulka, Hannah Griebler, Jana Drozenova, Katerina Mojzisova, Ann Vankerckhoven, Jan Laco, Ales Ryska, Pavel Dundr, Roman Kocian, David Cibula, Tomas Brtnicky, Petr Skapa, Francis Jacob, Marek Kovar, Ivan Praznovec, Iain A. McNeish, Michal J. Halaska, Lukas Rob, An Coosemans, Sandra Orsulic, Lorenzo Galluzzi, Radek Spisek, Jitka Fucikova

**Affiliations:** 1 Sotio Biotech, Prague, Czech Republic.; 2 Department of Immunology, Charles University, Second Faculty of Medicine and University Hospital Motol, Prague, Czech Republic.; 3 Department of Pathology, Third Faculty of Medicine and University Hospital Kralovske Vinohrady, Prague, Czech Republic.; 4 The Fingerland Department of Pathology, Charles University, Faculty of Medicine in Hradec Kralove and University Hospital Hradec Kralove, Hradec Kralove, Czech Republic.; 5 Department of Pathology, First Faculty of Medicine, Charles University and General University Hospital in Prague, Prague, Czech Republic.; 6 Department of Gynaecology, Obstetrics and Neonatology, General University Hospital in Prague, First Faculty of Medicine, Charles University, Prague, Czech Republic.; 7 Department of Gynecology and Obstetrics, First Faculty of Medicine, Charles University, University Hospital Bulovka, Prague, Czech Republic.; 8 Department of Pathology and Molecular Medicine, Second Faculty of Medicine, Charles University and University Hospital Motol, Prague, Czech Republic.; 9 Ovarian Cancer Research, Department of Biomedicine, University Hospital Basel and University of Basel, Basel, Switzerland.; 10 Laboratory of Tumor Immunology, Institute of Microbiology of the Czech Academy of Sciences, Prague, Czech Republic.; 11 Department of Gynecology and Obstetrics, Charles University, Faculty of Medicine and University Hospital Hradec Kralove, Hradec Kralove, Czech Republic.; 12 Department of Surgery and Cancer, Ovarian Cancer Action Research Centre, Imperial College London, London, United Kingdom.; 13 Department of Gynecology and Obstetrics, Charles University, Third Faculty of Medicine and University Hospital Kralovske Vinohrady, Prague, Czech Republic.; 14 Laboratory of Tumor Immunology and Immunotherapy, Department of Oncology, Leuven Cancer Institute, KU Leuven, Leuven, Belgium.; 15 Department of Obstetrics and Gynecology, David Geffen School of Medicine, University of California, Los Angeles, Los Angeles, California.; 16 Department of Veterans Affairs, Greater Los Angeles Healthcare System, Los Angeles, California.; 17 Department of Radiation Oncology, Weill Cornell Medical College, New York, New York.; 18 Sandra and Edward Meyer Cancer Center, New York, New York.; 19 Caryl and Israel Englander Institute for Precision Medicine, New York, New York.

## Abstract

**Purpose:**

Patients with high-grade serous ovarian carcinoma (HGSOC) are virtually insensitive to immune checkpoint inhibitors (ICI) employed as standalone therapeutics, at least in part reflecting microenvironmental immunosuppression. Thus, conventional chemotherapeutics and targeted anticancer agents that not only mediate cytotoxic effects but also promote the recruitment of immune effector cells to the HGSOC microenvironment stand out as promising combinatorial partners for ICIs in this oncological indication.

**Experimental Design:**

We harnessed a variety of transcriptomic, spatial, and functional assays to characterize the differential impact of neoadjuvant paclitaxel-carboplatin on the immunological configuration of paired primary and metastatic HGSOC biopsies as compared to neoadjuvant chemotherapy (NACT)-naïve HGSOC samples from five independent patient cohorts.

**Results:**

We found NACT-driven endoplasmic reticulum stress and calreticulin exposure in metastatic HGSOC lesions culminates with the establishment of a dense immune infiltrate including follicular T cells (T_FH_ cells), a prerequisite for mature tertiary lymphoid structure (TLS) formation. In this context, TLS maturation was associated with an increased intratumoral density of ICI-sensitive TCF1^+^PD1^+^ CD8^+^ T cells over their ICI-insensitive TIM-3^+^PD1^+^ counterparts. Consistent with this notion, chemotherapy coupled with a PD1-targeting ICI provided a significant survival benefit over either therapeutic approach in syngeneic models of HGSOC bearing high (but not low) tumor mutational burden.

**Conclusions:**

Altogether, our findings suggest that NACT promotes TLS formation and maturation in HGSOC lesions, *de facto* preserving an intratumoral ICI-sensitive T-cell phenotype. These observations emphasize the role of rational design, especially relative to the administration schedule, for clinical trials testing chemotherapy plus ICIs in patients with HGSOC.

*See related commentary by Bravo Melgar and Laoui, p. 10*

**Translational Relevance**

Ovarian carcinoma is largely resistant to immune checkpoint inhibitors (ICI), reflecting not only a limited amount of tumor‐infiltrating immune effector cells but also the ability of ovarian carcinoma cells to establish robust intratumoral immunosuppression. Thus, strategies aimed at converting immunologically “cold” ovarian carcinoma lesions into their “hot” counterparts stand out as promising combinatorial partners for ICI-based immunotherapy in this oncological indication. Here, we demonstrate that neoadjuvant chemotherapy (NACT)‐driven endoplasmic reticulum stress in metastatic high-grade serous carcinoma (HGSOC) lesions culminates with the establishment of a dense immune infiltrate and tertiary lymphoid structures (TLS) formation. In this context, TLS maturation is associated with an increased intratumoral density of ICI-sensitive TCF1^+^PD1^+^ CD8^+^ T cells over their ICI-insensitive TIM‐3^+^PD1^+^ counterparts. These observations emphasize the role of accurately designed clinical studies involving a diversified immune-monitoring program to unlock the full therapeutic potential of modern ICI‐based combinatorial regimens against metastatic HGSOC.

## Introduction

High-grade serous ovarian carcinoma (HGSOC) is the most common ovarian neoplasm (1). Despite recent progress in clinical HGSOC management, treatment remains challenging and survival rates have only modestly improved over the past four decades (2, 3). Such an unfavorable disease outcome is mainly linked to the ability of ovarian cancer cells to disseminate via the peritoneum and colonize omental fat deposits at early disease stages, ultimately establishing an immunosuppressed tumor microenvironment (TME) that enables rapid disease progression and resistance to therapy at metastatic sites (4, 5). Standard-of-care (SOC) HGSOC management comprises cytoreductive surgery coupled with platinum-taxane doublet chemotherapy (2), an approach that enables complete remission in most patients, but (most often) ultimately selects for chemoresistant disease leading to treatment failure and relapse (6).

The widespread clinical success of immune checkpoint inhibitors (ICIs; refs. 7, 8) created enormous expectations around the possibility that ICIs would mediate clinically relevant activity in patients with HGSOC (3, 9). However, women with HGSOC are virtually insensitive to ICIs (3, 10, 11), both when used as standalone therapeutic agents and when combined upfront with SOC therapeutic approaches, reflecting not only the limited amount of tumor-infiltrating immune effector cells (12–14) but also the ability of HGSOC cells to establish robust intratumoral immunosuppression (3, 15, 16). Thus, strategies aimed at converting immunologically “cold” HGSOC lesions into their “hot” counterparts, encompassing abundant infiltration by mature dendritic cells (DC), T_H_1-polarized helper T CD4^+^ cells, and CD8^+^ cytotoxic T lymphocytes (CTL), stand out as promising combinatorial partners for ICI-based immunotherapy in this oncological indication (17–21).

At least in some settings, a TME that actively recruits and activates immune cells can be established through therapeutic measures eliciting immunogenic cell stress and death, that is, strategies that alter cancer cell homeostasis in a way that engages the host immune system to unleash a tumor-specific immune response associated with effector and memory features (22, 23). Among other elements, such an immunogenic response relies on antigenicity, that is, the ability of cancer cells to express antigens not covered by central tolerance on MHC class I molecules, and adjuvanticity, that is, the capacity of stressed and dying malignant cells to engage innate and adaptive immune effectors via immunostimulatory factors cumulatively known as damage-associated molecular patterns (DAMPs; refs. 24, 25).

Accumulating preclinical and clinical evidence indicates that some cytotoxic chemotherapeutics (notably anthracyclines, some platinum derivatives, and taxanes), radiation therapy (at least when used according to specific dose and fractionation protocols), and targeted anticancer agents (including, but not limited to, tumor-targeting monoclonal antibodies and tyrosine kinase inhibitors) can elicit immunogenic cell stress and death across a variety of tumor types correlating with (at least partially restored) sensitivity to ICIs (26–28). That said, while preclinical data suggest that NACT may restore ICI sensitivity in syngeneic models of HGSOC, such combinatorial regimens have failed to provide clinical benefits over SOC disease management so far (29–31). At least in part, this may reflect the fact that metastatic HGSOC lesions forming soon after the establishment of the primary disease site might exhibit an immune contexture that is not permissive for the ICI-driven activation of anticancer immunity, a possibility that has not been investigated in detail (32–35).

Here, we harnessed a variety of transcriptomic, spatial, and functional assays to characterize the differential impact of NACT on the microenvironment of paired primary and metastatic HGSOC biopsies as compared to NACT-naïve HGSOC samples from five independent patient cohorts. We found that metastatic HGSOC samples exhibiting signs of increased adjuvanticity in the context of NACT are also characterized by an increased density of effector CD8^+^ CTLs, CD20^+^ B cells, follicular helper T (T_FH_) cells, and mature tertiary lymphoid structures (TLS). In this context, TLS maturation correlated with an increased abundance of ICI-sensitive TCF1^+^PD1^+^ CD8^+^ T cells over their ICI-insensitive TIM-3^+^PD1^+^ counterparts. In line with these observations, platinum-taxane chemotherapy positively interacted with an ICI targeting programmed cell death 1 (PDCD1, best known as PD1) in a syngeneic preclinical mouse model of ovarian cancer bearing high (but not low) tumor mutational burden.

## Materials and Methods

### Clinical samples and patient characteristics

All tissue samples and health-related data in our study were collected after ethical review and approval of the Ethics Committee listed below.

#### Study cohort 1

A retrospective series of 130 formalin-fixed, paraffin-embedded (FFPE) paired primary (*n* = 70) and metastatic (*n* = 60) tumor samples were obtained from 70 FIGO stage III + IV patients with HGSOC who underwent primary surgery in the absence of NACT between 2008 and 2014 at University Hospital Hradec Kralove (Supplementary Fig. S1). Baseline characteristics of these patients are summarized in Supplementary Table S1. Pathology staging was performed according to the eighth TNM classification from 2017, and histologic types were determined according to the current WHO classification (36). Written informed consent was obtained from patients before inclusion in the study. Data on long-term clinical outcomes were obtained retrospectively by interrogation from municipality registers or families. The protocol was approved by the local Ethical Committee (201607S14P).

#### Study cohort 2

A retrospective series of 80 FFPE primary (*n* = 40) and metastatic (*n* = 40) tumor samples were obtained from 40 FIGO stage III + IV patients with HGSOC who underwent primary surgery after three cycles of neoadjuvant paclitaxel-carboplatin–based chemotherapy between 2008 and 2014 at University Hospital Hradec Kralove (Supplementary Fig. S1). Baseline characteristics of these patients are summarized in Supplementary Table S1. Pathology staging was performed according to the eighth TNM classification from 2017, and histologic types were determined according to the current WHO classification (36). Data on long-term clinical outcome were obtained retrospectively by interrogation from municipality registers or families. The protocol was approved by the local Ethical Committee (201607S14P).

#### Study cohort 3

An additional series of primary (*n* = 39) and metastatic (*n* = 20) samples were prospectively collected from 39 patients with FIGO stage III + IV HGSOC who underwent primary surgery in the absence of NACT at University Hospital Kralovske Vinohrady (Supplementary Fig. S1). This study was conducted in accordance with the Declaration of Helsinki and the protocol was approved by the local Ethical Committee (Progress UK, Q40/11). Written informed consent was obtained from patients before inclusion in the study. Baseline characteristics of these patients are summarized in Supplementary Table S2.

#### Study cohort 4

A prospective series of primary (*n* = 19) and metastatic (*n* = 19) tumor samples were obtained from 19 patients with FIGO stage III + IV HGSOC who underwent primary surgery after three cycles of neoadjuvant paclitaxel-carboplatin–based chemotherapy between 2008 and 2014 at the University Hospital Kralovske Vinohrady (Supplementary Fig. S1). Baseline characteristics of these patients are summarized in Supplementary Table S2. Pathology staging was performed according to the eighth TNM classification from 2017, and histologic types were determined according to the current WHO classification (36). Data on long-term clinical outcome were obtained retrospectively by interrogation from municipality registers or families. The protocol was approved by the local Ethical Committee.

#### Study cohort 5

A prospective series of tissue samples were obtained from 11 patients with HGSOC who underwent primary laparoscopy and interval debulking surgery after three cycles of platinum-taxane doublet. Baseline characteristics of these patients are summarized and published previously (37). The protocol was approved by the local Ethical Committee.

### Immunohistochemistry and immunofluorescence analyses

Immunostaining with antibodies specific for CD8, CD20, and lysosomal associated membrane protein 3 (LAMP3; best known as DC-LAMP) was performed according to conventional protocols (38). Briefly, tumor specimens were fixed in neutral buffered 10% formalin solution and embedded in paraffin as per standard procedures. In brief, 4-µm-thick tissue sections were deparaffinized and rehydrated in a descending alcohol series (100%, 96%, 70%, and 50%), followed by antigen retrieval with Target Retrieval Solution (Leica) in EDTA pH 8.0 (for CD8, CD20, DC-LAMP) in a preheated water bath (97°C, 30 minutes). Sections were allowed to cool down to RT for 30 minutes. Endogenous peroxidase or alkaline phosphatase was blocked with 3% H_2_O_2_ or blocking solution Bloxall (Vector), respectively, for 10 to 15 minutes. Thereafter, sections were treated with Protein Block (DAKO) for 15 minutes and incubated with primary antibodies, followed by the revelation of enzymatic activity (Supplementary Table S3). Sections were counterstained with hematoxylin (DAKO) for 30 seconds. Images were acquired using a Leica Aperio AT2 scanner (Leica). Immunofluorescence panel 1 (CD4, CD8, CD20, CD21, CD23, DC-LAMP, and GZMB) were performed according to a previously published protocol (Supplementary Table S3; ref. 12). Briefly, 4-µm-thick FFPE tissue sections were deparaffinized and rehydrated in a descending alcohol series (100%, 96%, 70%, and 50%), followed by antigen retrieval with Target Retrieval Solution (Leica) in EDTA pH 8.0 with a heated water bath (97°C, 30 minutes). Sections were allowed to cool down to room temperature (RT) for 30 minutes, then treated with Signal Enhancer (Thermo Fisher Scientific) for 30 minutes and Blocking Buffer (Thermo Fisher Scientific) for 1 hour. The DC-LAMP-specific antibody (Dendritics, 1:350) was incubated overnight at 4°C, the CD8-specific antibody (Abcam, 1:60) for 90 minutes at RT, the CD20-specific antibody (Dako, 1:300) for 1 hour at RT. Thereafter, slides were incubated with appropriate HRP Polymer secondary antibodies for 1 hour at RT, followed by Tyramide Signal Amplification (Thermo Fisher Scientific; Supplementary Table S3). Finally, sections were treated with TrueBlack Lipofuscin Autofluorescence Quencher (Biotium) for 30 seconds and mounted with ProLong Gold Antifade Reagent containing DAPI (Thermo Fisher Scientific; Supplementary Table S3). Staining specificity was determined using appropriate isotype controls. Images of whole tumor sections were acquired using a Leica Aperio AT2 scanner (Leica). Thereafter the same sections were restained with antibodies specific for CD4, CD21, and CD23 using sequential IHC protocol (Supplementary Table S3; see Supplementary Materials and Methods; ref. 39). Between every staining step, the slides were scanned and the final image was composed by the HALO10 software (Indica Labs) using the deconvolution and registration algorithm.

Immunofluorescence panel 2 with antibodies specific for CD8, PD1, CD4, FoxP3, CD20, GZMB, CD68, CD274 (best known as PD-L1) was performed according to the manufacturer’s instructions (ULTIVUE; Supplementary Table S3). Thereafter the same sections were restrained with antibodies specific for TCF1, CD57, TIM-3, and PanCyto using sequential immunohistochemistry (IHC) protocol. Further details are provided in Supplementary Materials and Methods. Immunofluorescence panel 3 (CXCR5, PD1, FoxP3, CD23, CD20, and CD4) was performed using sequential IHC (Supplementary Table S3; for full details see Supplementary Materials and Methods). Sections were treated with Normal Horse serum 2.5% (Vector) for 20 minutes and incubated with primary antibodies anti-human CXCR5, followed by the revelation of enzymatic activity (Supplementary Table S3). Sections were counterstained with hematoxylin (DAKO) for 2 minutes. Images were acquired using a Leica Aperio AT2 scanner (Leica). Thereafter the same sections were restrained with antibodies specific for PD1, FoxP3, CD23, CD20, and CD4 using sequential IHC protocol (Supplementary Table S3). Between every staining step the slides were scanned, and the final image was composed by the HALO10 software (Indica Labs) using the registration algorithm.

### Flow cytometry analyses

As previously described, total live mononuclear cells were isolated from fresh tumor specimens (Supplementary Fig. S2; ref. 12). The phenotype of CD8^+^ T cells was assessed at day 2 using panel of fluorescent extracellular and intracellular primary antibodies or appropriate isotype controls for 20 minutes at 4°C in the dark, followed by washing and acquisition on a Fortessa flow cytometer (BD Biosciences; Supplementary Table S4). Flow cytometry data were analyzed with the FlowJo software (BD Biosciences; Supplementary Fig. S2).

### Quantitative evaluation of TLSs and cell densities

Only TLS made up of more than 50 cells were included in the analysis (Supplementary Fig. S3; ref. 12). Tumor samples with cellular aggregates that contain more than 50 cells on hematoxylin and eosin (H&E) were further analyzed by IHC using anti-CD20 and -CD23 antibodies. In the absence of CD21^+^ and CD23^+^ positivity, the TLS was identified as early TLS (Supplementary Fig. S3A). In addition, eTLS were defined as aggregates of minimum size of 250 μm, with majority of cells being CD20^+^ B cells in close proximity contacts (min 3 μm), with presence of CD4^+^, CD8^+^ T cells. TLS were defined as “mature” when at least one CD21^+^ and CD23^+^ dendritic cell was detected in the TLS (Supplementary Fig. S3B and S3C), further using immunofluorescence panel 1. Cell density (cells/mm^2^) for CD8^+^ T cells, DC-LAMP^+^ DCs, CD20^+^ B cells, CD4^+^ T cells, CD68^+^CD163^+^ TAMs, CXCR5^+^PD1^+^FoxP3^−^CD4^+^ T_FH_ cells, TCF1^+^PD1^+^CD8^+^ T cells, and TIM-3^+^PD1^+^CD8^+^ T cells was quantified in whole tumor sections by the HALO10 software (Indica Labs) using the HighPlex FL 4.1.0.3 and classifier algorithm. Quantitative assessments were performed by three independent investigators (T. Lanickova, L. Kasikova, J. Fucikova) and independently reviewed by either of the two expert pathologists (J. Laco, A. Ryska).

### Next-generation sequencing data analysis

As previously described (5), hierarchical clustering analysis was conducted for differentially expressed genes using the ComplexHeatmap package in R, based on the Euclidean distance and ward.D2 clustering method. The MCP-counter R package was used to estimate the abundance of tissue-infiltrating immune cell populations on bulk RNA-seq data (40).

### scRNA-seq sample preparation

Prospective tumor samples were from 11 patients with HGSOC at the time of laparoscopy (prior NACT) and interval debulking surgery (after NACT). Detailed clinical information is presented within original study (37). Tumor specimens were incubated in a mixture of collagenase and hyaluronidase after surgery to obtain single-cell suspension. The viability of the cell suspensions ranged from 65% to 94%. Single-cell RNA sequencing (scRNA-seq) libraries were prepared with the Chrominum Single-Cell 3′Reagent kit v. 2.0 (10× Genomics) and sequenced on Illumina HiSeq 4000, HiSeq 2500, and NovaSeq 6000 instruments as described in detail previously (37).

### Preprocessing scRNAseq data

Cellular annotation file and UMI counts were downloaded from GSE165897. R package Seurat (v 5.0.1) was applied to run cell quality control and normalize the gene expression. Low-quality cells were removed according to the following criteria: nFeature <500 or nFeature >7,000; mitochondrial gene content >15%. After data filtering, the scRNA-seq data were normalized by NormalizeData function. Principal component analysis (PCA) was constructed based on the scaled data with the top 2,000 highly variable genes. Reciprocal PCA (RPCA) analysis was applied for batch effect removal and primary clustering. SingleR (Version: 2.0.0) package was utilized for cell cluster annotation, while the reference was loaded from the celldex package BlueprintEncodeData (41). Differential gene expression (DGE) analysis was performed using pseudobulk conversion (AggregateExpression function), followed by a DESeq2 analysis in the Seurat package. To identify T-cell subtypes, we projected CD8^+^ T cells into reference single-cell atlases using ProjectTILs (42). All codes employed or generated during the analysis are available publicly at GitHub.

### Mice

Female C57BL/6 mice, aged 8 to 12 weeks were from the Institute of Microbiology, Czech Academy of Sciences. Housing was in individually ventilated cages. Food and water were provided *ad libitum*, Teklad Global 18% Protein Rodent Diet, irradiated; water in sterile prefilled bottles. Dark/Light cycle on a 12-hour automated schedule; temperature 21°C ± 2°C; and humidity 50% ± 10%. Animals were randomly assigned to experimental groups; no blinding was performed during these experiments.

### Mouse tumor models

SO1 (kindly provided by Dr. Sandra Orsulic, University of California, Los Angeles) murine ovarian cancer cells were cultured in the Dulbecco’s Modified Eagle Medium (DMEM; Sigma) supplemented with 10% fetal bovine serum (FBS; Gibco), antibiotics (100 U mL^−1^ penicillin sodium and 100 μg mL streptomycin sulfate, (Gibco). ID8 (kindly provided by prof. Ian A. McNeish, Imperial College London) murine ovarian cancer cells were cultured in DMEM (Sigma) supplemented with 4% fetal bovine serum (FBS, Sigma Aldrich), 1% Insulin -Transferin-Selenium (ITS; Sigma) and antibiotics. All cells were cultured at 37°C with 5% CO_2_ before being harvested and suspended in serum-free DMEM (Sigma) for tumor injection. All cell lines were routinely checked for mycoplasma spp. contamination by the PCR-based LookOut Mycoplasma PCR Detection kit (Millipore Sigma). 1 × 10^6^ SO1 and 6 × 10^6^ ID8 tumor cells were mixed at a 1:1 ratio with cold Cultrex BME (Biotechne) and injected intraperitoneally into 8 to 12 weeks old C57BL/6 mice. For all mice experiments, animals were routinely monitored for disease progression and overt toxicity such as >20% decline in body weight, lethargy, immobility, fur ruffling, and fur loss. Mice were euthanized upon experimental endpoint. Mice reached a humane endpoint and were humanely euthanized upon >50% body weight increase due to ascites formation or institutional compassionate euthanasia criteria were met. Maximal tumor size/burden was not exceeded. Animal experiments strictly followed a protocol approved by the Institutional Animal Care and Use Committee of the Academy of Sciences of the Czech Republic and conducted in compliance with the local and European guidelines.

### Statistical analysis

Survival analyses were estimated by Cox proportional hazard regressions and the Kaplan–Meier method, where differences between the groups of patients were calculated using the log-rank test. For log-rank tests, the prognostic value of continuous variables was assessed using median cutoff of intratumoral immune cell densities, TLS number, and calreticulin (CALR) score. The Mann–Whitney test was used to compare the density of tumor-infiltrating cells among patient groups. The Fisher exact test was used to compare patient distribution across subgroups. The enrichGo function from ClusterProfiler was used to identify enriched Gene Ontology (GO) terms based on hypergeometric distribution. *P* values were adjusted for multiple comparisons using the Benjamini–Hochberg correction. All analyses were performed with Prism 8.4.2 (GraphPad) and R software V.4.2.2 (http://www.r-project. org/). *P* values <0.05 were considered statistically significant.

### Data availability

The data generated in this study are available upon request to the corresponding author.

All analyses reported in this study used the statistical software R (v.4.2.2). All codes employed or generated during the current study are available publicly at https://github.com/HenslerM/Lanickova_CCR2024.

## Results

### NACT promotes adjuvanticity and immune cell recruitment in metastatic HGSOC

**Figure 1.**
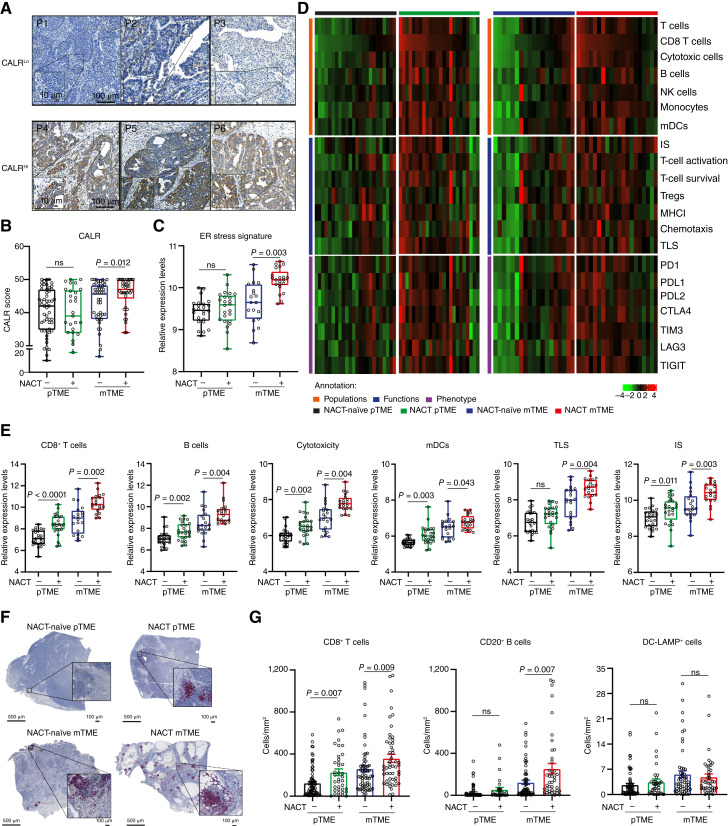
Immunomodulation by NACT in metastatic HGSOC. **A,** Representative images of CALR immunostaining in CALR^Lo^ and CALR^Hi^ patients. Scale bars, 10 and 100 µm. **B,** CALR expression levels determined by immunostaining and (**C**) ER stress signature level [expression level of DNA damage inducible transcript 3 (DDIT3, best known as CHOP), heat shock protein family A (Hsp70) member 5 (HSPA5, best known as BIP), and heat shock protein 90 beta family member 1 (HSP90B1)] as determined by RNA-seq in pTME and mTME HGSOC with/without NACT. Box plots: lower quartile, median, upper quartile; whiskers, minimum, maximum. Statistical significance was calculated by two-sided Mann–Whitney test. *P* values are indicated. **D,** Supervised hierarchical clustering of gene signatures related to immune populations (orange), immune functions (blue) and immune phenotype (purple) as determined by RNA-seq data from pTME and mTME HGSOC tumor samples with/without NACT. IS, immunosuppression; mDCs, myeloid dendritic cells; NK cells, natural killer cells; TLS, tertiary lymphoid structures. **E,** Gene expression signature associated with CD8^+^ T cells, B cells, cytotoxicity, mDCs, TLS, and immunosuppression as determined on RNA-seq data from pTME and mTME HGSOC with/without NACT. Box plots: lower quartile, median, upper quartile; whiskers, minimum, maximum. Statistical significance was calculated by two-sided Mann–Whitney test. *P* values are indicated. **F,** Representative image of CD20/DC-LAMP double immunostaining. Scale bars, 500 and 100 µm. **G,** Density of CD8^+^ T cells, CD20^+^ B cells, and DC-LAMP^+^ cells as determined by immunostaining in pTME and mTME HGSOC samples with/without NACT. Mean and SEM are shown. Statistical significance was calculated by two-sided Mann–Whitney test. *P* values are indicated. ns, not significant.

To investigate the adjuvanticity of NACT in the HGSOC setting, paired primary TME (pTME), and metastatic TME (mTME), samples from retrospective series of patients with stage III and IV HGSOC who did not receive NACT (study cohort 1; *n* = 70) or did so (study cohort 2; *n* = 40) were analyzed for the expression of the endoplasmic reticulum (ER) chaperone and DAMP CALR by IHC (Fig. 1A and B). While CALR was expressed at heterogeneous levels across pTME and mTME samples from both study cohorts (Fig. 1B), and expression did not differ between pTME biopsies from patients who had received NACT or not, we observed significantly increased CALR levels in mTME samples obtained after NACT as compared to their NACT-naïve counterparts, an observation that was true for malignant, but not immune, cells (Fig. 1B; Supplementary Fig. S4). Such an increase correlated with transcriptional signs of ER stress, as computed from the mRNA levels of DNA damage inducible transcript 3 (DDIT3, best known as CHOP), heat shock protein family A (Hsp70) member 5 (HSPA5, best known as BIP), and heat shock protein 90 beta family member 1 (HSP90B1; Fig. 1C).

Inspired by these findings, we set to harness RNAseq to compare the transcriptomic profile of 48 paired pTME and mTME from both study cohort 1 and 2 (Fig. 1D). DGE analysis followed by gene set enrichment analysis based on GO terms mainly identified transcriptional signatures linked to the immune response, lymphocyte trafficking, B-cell receptor signaling, and immune system regulation as overrepresented in the pTME and mTME of patients receiving NACT versus their NACT-naïve counterparts (Fig. 1D; Supplementary Table S5). Next, we employed “metagene” markers (as per the MCP counter method; ref. 40) to estimate the relative abundance of different immune cell populations and immunological processes (Fig. 1E; Supplementary Fig. S5). In line with whole-transcriptome findings (Fig. 1D), NACT-treated pTME and mTME samples were enriched for gene sets associated with CD8^+^ T cells (pTME: *P* < 0.0001; mTME: *P* = 0.002), B cells (pTME: *P* = 0.002; mTME: *P* = 0.004), cytotoxicity (pTME: *P* = 0.002; mTME: *P* = 0.004), myeloid DCs (pTME: *P* = 0.003; mTME: *P* = 0.043), TLSs (pTME: ns; mTME: *P* = 0.004), and immunosuppression (pTME: *P* = 0.011; mTME: *P* = 0.003), as compared to their NACT-naïve counterparts (Fig. 1E). To validate these data with another technological approach, we analyzed the immune infiltrate of pTME and mTME samples from both study cohorts by IHC (Fig. 1F). Confirming transcriptomic observations, we detected a higher density of CD8^+^ CTLs in samples from NACT-treated patients versus their NACT-naïve counterparts, in both the pTME (*P* = 0.007) and the mTME (*P* = 0.009; Fig. 1G). A similar observation was true for the density CD20^+^ B cells, only in metastatic samples (*P* = 0.007), but not for the intratumoral abundance of DC-LAMP^+^ DCs (Fig. 1G). In line with previous findings from us and others (14, 43–46), an increased density of CD8^+^ T cells with the pTME and mTME of NACT-naïve patients with stage III-IV HGSOC correlated with improved overall survival (OS) as determined by Kaplan–Meier survival curves (Supplementary Fig. S6A and S6B) and univariate Cox analyses (Table 1). Conversely, we failed to observe virtually any prognostic impact for intratumoral CD8^+^ T cells in patients with late-stage HGSOC receiving NACT, possibly due to the limited number of patients included in this retrospective analysis (Supplementary Fig. S6C and S6D).

**Table 1.** Univariate Cox proportional hazard analyses.

Variable	Cohort 1			Cohort 2		
		HR (95%confidence interval)	*P* value		HR (95%confidence interval)	*P* value
Age		1.02 (1.00–1.05)	0.107		1.01 (0.98–1.05)	0.531
CA125		1 (1.00–1.00)	0.813		1 (1.00–1.00)	0.719
Debulking	R0	1		R0	1	
	R1	0.71 (0.21–2.43)	0.581	R1	1.06 (0.35–3.25)	0.912
	R2	1.21 (0.67–2.21)	0.530	R2	1.28 (0.63–2.62)	0.491
pTME CALR		0.98 (0.94–1.01)	0.137		1.01 (0.97–1.04)	0.780
pTME CD8		0.57 (0.91–1.03)	0.035		1.00 (1.00–1.00)	0.227
pTME DCLAMP		0.98 (0.95–1.02)	0.369		1.00 (0.93–1.08)	0.974
pTME CD20		0.3 (0.07–1.25)	0.098		1.44 (0.61–3.37)	0.402
pTME M2-like TAMs		1 (0.99–1.01)	0.835		0.97 (0.76–1.25)	0.835
pTME mTLS		0.97 (0.95–1.00)	0.025		0.98 (0.95–1.02)	0.336
mTME CALR		0.95 (0.92–0.99)	0.137		0.97 (0.89–1.06)	0.528
mTME CD8		0.95 (0.90–1.01)	0.041		1.00 (1.00–1.00)	0.988
mTME DCLAMP		0.95 (0.90–1.01)	0.089		1.04 (0.95–1.13)	0.387
mTME CD20		0.96 (0.89–1.03)	0.243		1.06 (0.65–1.74)	0.815
mTME M2-like TAMs		1 (1.00–1.01)	0.484		0.98 (0.91–1.06)	0.645
mTME mTLS		0.99 (0.96–1.02)	0.042		0.98 (0.96–1.00)	0.046

Abbreviations: CALR, calreticulin; mTLS, tertiary lymphoid structures; TAMs, tumor‐associated macrophages.

These findings suggest that NACT appears to preferentially increase the adjuvanticity of metastatic HGSOC, correlating with signs of increased tumor infiltration by immune effector cells including CD8^+^ CTLs and CD20^+^ B cells but not DC-LAMP^+^ DCs.

### NACT promotes clinically relevant TLS maturation in the metastatic HGSOC environment

**Figure 2.**
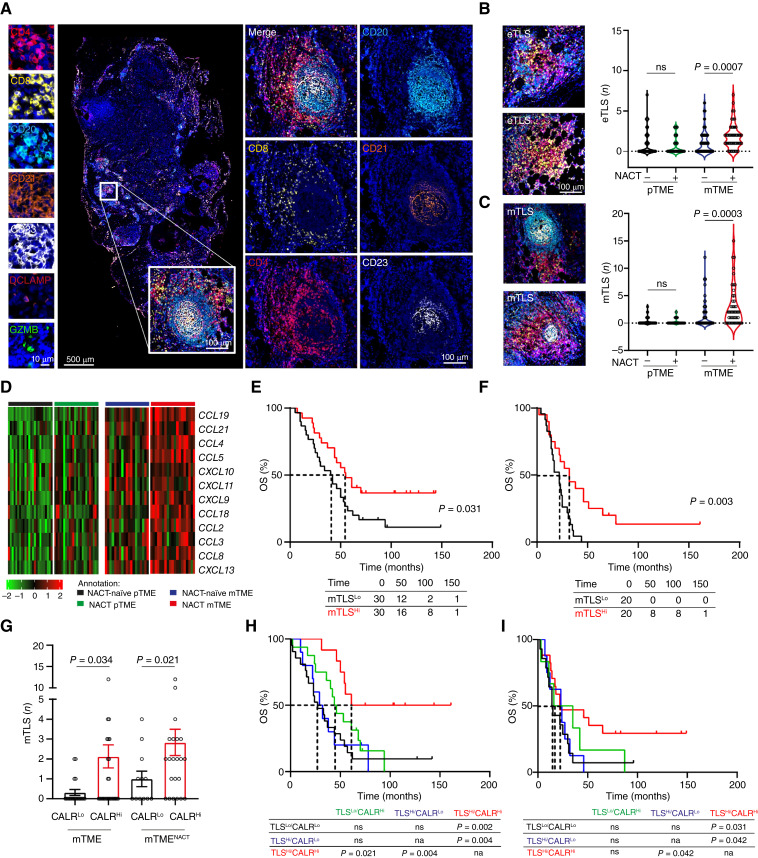
NACT-mediated adjuvanticity positively impacts clinically relevant TLS maturation in metastatic HGSOC. **A,** Representative image of immunofluorescence of CD4, CD8, CD20, CD21, CD23, DC-LAMP, and GZMB staining (immunofluorescence panel 1). Scale bars, 10, 100 and 500 µm. **B** and **C,** Distribution of early TLS (eTLS; **B**) and mature TLS (mTLS; **C**) across pTME and mTME HGSOC tumor samples with/without NACT. Statistical significance was calculated by two-sided Mann–Whitney test. *P* values are indicated. **D,** Supervised hierarchical clustering of TLS-relevant gene signature (*CCL2*, *CCL3*, *CCL4*, *CCL5*, *CCL8*, *CCL18*, *CCL19*, *CCL21*, *CXCL9*, *CXCL10*, *CXCL11*, and *CXCL13*) across pTME and mTME HGSOC tumor samples with/without NACT. **E** and **F,** Overall survival (OS) of 60 (**E**) and 40 chemo-naïve and treated patients with mHGSOC (**F**), respectively (study cohort 1 and 2) based on median stratification of total mTLS. Survival curves were estimated by the Kaplan–Meier method, and differences between groups were evaluated using log-rank test. Number of patients at risk and *P* values are reported. **G,** Number of mTLS across patients with CALR^Lo^ and CALR^Hi^ mHGSOC with/without NACT as determined by median stratification. Mean and SEM are shown. Statistical significance was calculated by two-sided Mann–Whitney test. *P* values are indicated. **H** and **I,** OS of 60 and 40 patients with chemo-naïve and treated mHGSOC (study cohorts 1 and 2), upon stratification based on median frequency of mTLS and expression of CALR. Survival curves were estimated by the Kaplan–Meier method, and differences between groups were evaluated using the log-rank test.

Inspired by our observations linking NACT to an increased density of CD8^+^ CTLs and CD20^+^ B cells in mTME biopsies from patients with HGSOC, we set to assess the frequency of early TLS (eTLS) and mature TLS (mTLS) using immunofluorescence microscopy based on CD4, CD8, CD20, CD21, CD23, DC-LAMP, and GZMB (Fig. 2A and B; Supplementary Fig. S3A–S3C). TLS with primary (CD21^+^ DCs) and/or secondary (CD21^+^CD23^+^ DCs) follicles were defined as mTLS. Supporting our previous observations based on pTME biopsies from patients with HGSOC (12), we failed to detect enrichment in eTLS and mTLS when comparing NACT-treated pTME samples to their NACT-naïve counterparts (Fig. 2B and C). In contrast, both eTLSs and mTLSs were significantly increased within the mTME of patients with NACT-treated HGSOC as compared to NACT-naïve individuals (Fig. 2B and C). In line with this notion, mTME (but not pTME) samples from patients with HGSOC receiving NACT exhibited an enrichment in a TLS-relevant chemokine gene signature (*CCL2*, *CCL3*, *CCL4*, *CCL5*, *CCL18*, *CCL19*, *CXCL9*, and *CXCL13*) as compared to NACT-naïve mTME biopsies (Fig. 2D; Supplementary Fig. S7).

As mTLSs are significantly more abundant in the mTME than in the pTME of HGSOC (12), we set to assess the prognostic value of mTLS abundance in the mTME of patients with HGSOC who received NACT or not, upon stratification of Study cohort 1 and 2 by median mTLS. We found that patients with higher-than-median mTLSs (TLS^Hi^) had prolonged OS as compared to their TLS^Lo^ counterparts in both study cohorts (study cohort 1: *P* = 0.031; study cohort 2: *P* = 0.003), as per Kaplan–Meier survival analysis (Fig. 2E and F), but only in patients receiving NACT these data were confirmed by univariate Cox regression (Table 1).

Intriguingly, in mTME samples from both study cohort 1 and 2, mTLSs were more abundant in the presence of higher-than-median CALR (CALR^Hi^) expression as determined by IHC (Fig. 2G). Consistent with this, stratifying study cohort 1 and 2 based on median mTLS abundance and median CALR expression documented significantly improved OS for patients with a TLS^Hi^/CALR^Hi^ mTME over virtually all other patient subgroups, with the exception of NACT-naïve patients with a TLS^Lo^/CALR^Hi^ (Fig. 2H and I).

Altogether, these findings suggest that carboplatin-paclitaxel doublet NACT promotes eTLS and mTLS formation in the metastatic HGSOC microenvironment.

### B-cell profiling identifies *in situ* maturation and differentiation states associated with a high density of T_FH_ cells in metastatic HGSOC after NACT

**Figure 3.**
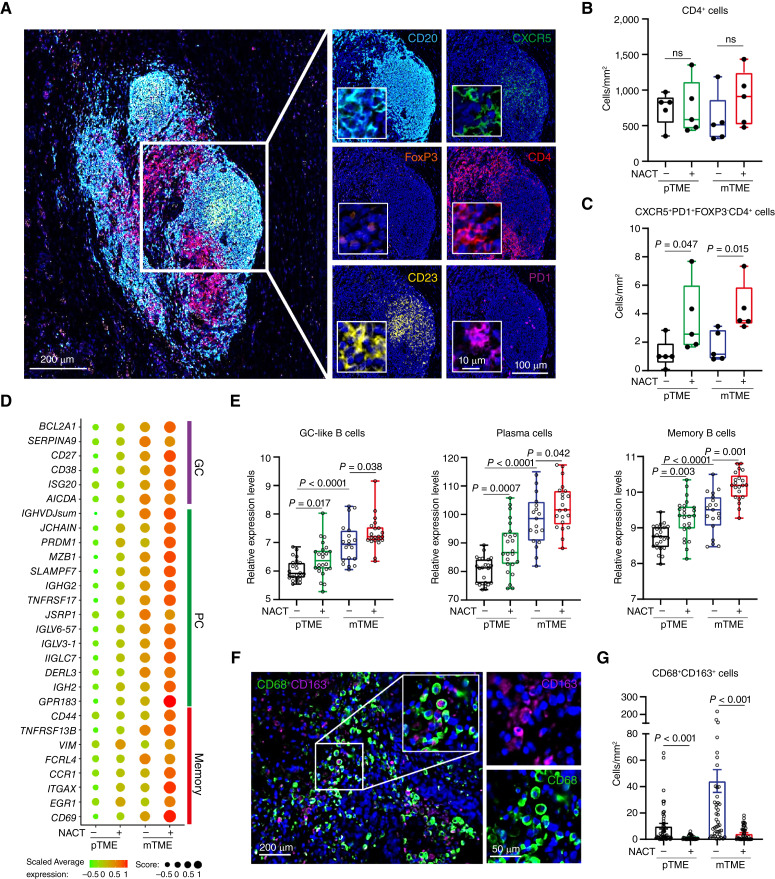
NACT-mediated adjuvanticity positively impacts the density of follicular T cells (T_FH_) and *in situ* activation of intratumoral B cells in mHGSOC. **A–C,** Representative image (**A**) and box plots showing the density of CD4^+^ cells (**B**) and CXCR5^+^PD1^+^FoxP3^−^CD4^+^ T_FH_ cells (**C**) in the pTME and mTME of chemo-naïve and treated HGSOC (study cohort 1 and 2). Box plots: lower quartile, median, upper quartile; whiskers, minimum, maximum. Statistical significance was calculated by the Mann–Whitney test. *P* values are indicated. **D** and **E,** Dot plot (**D**) and box plot (**E**) showing expression profile of gene signatures of B-cell subtypes, e.g., plasma cells (PC), germinal center (GC), and memory B cells within pTME and mTME HGSOC tumor samples with/without NACT. Box plots: lower quartile, median, upper quartile; whiskers, minimum, maximum. Statistical significance was calculated by two-sided Mann–Whitney test. *P* values are indicated. **F** and **G,** Representative image (**F**) and density of CD68^+^CD163^+^ TAMs in pTME and mTME of chemo naïve and treated HGSOC (**G**). Mean and SEM are shown. Statistical significance was calculated by two-sided Mann–Whitney test. *P* values are indicated.

As enhanced T_FH_ differentiation and density have been shown to induce TLSs associated with B-cell recruitment and activation, we next determined the density of CD4^+^CXCR5^+^PD1^+^FOXP3^−^ T_FH_ cells in biopsies from study cohort 1 and 2 using multiplex immunofluorescence (Fig. 3A). Supporting our previous observations (12), although the density of CD4^+^ T cells at large was virtually comparable in pTME and mTME samples irrespective of NACT (Fig. 3B), T_FH_ cells were significantly increased in NACT-treated mTME HGSOC samples as compared to their NACT-naïve counterparts (Fig. 3C), correlating with eTLS and mTLS development in this patient subgroup (Fig. 2B and C). As the formation of germinal centers (GC) is crucial for B cells differentiation within TLSs, we decided to dissect the localization of specific B-cell subsets (as previously defined by single-cell transcriptomic analyses of tonsillar B cells; ref. 47) with respect to TLS maturation in our HGSOC sample set. As expected, GC-like, plasma cell (PC) and memory B-cell signatures were enriched in the NACT-naïve mTME compared to its pTME counterpart, as well as in mTME samples from patients with HGSOC receiving NACT compared to their NACT-naïve counterparts (Fig. 3D and E). These findings are in line with the notion that mature TLSs support B-cell maturation, selection, and expansion *in situ*, culminating with the dissemination of mature PCs to the TME (48).

As we previously demonstrated that immunosuppressive tumor-associated macrophages (TAM) suppress tumor-targeting immunity in metastatic HGSOC (5, 16), we next determined the density of so-called “M2-like” CD163^+^CD68^+^ TAMs in pTME and mTME HGSOC samples from Study cohort 1 and 2 (Fig. 3F). In line with the notion that NACT positive impact HGSOC-targeting immune responses, we observed a significantly decreased density of CD68^+^ TAMs as well as M2-like CD163^+^CD68^+^ TAMs in both pTME and mTME samples from patients with HGSOC treated with NACT as compared to their NACT-naïve counterparts, an effect that was considerably more pronounced in the metastatic HGSOC microenvironment (Fig. 3G; Supplementary Fig. S8).

Altogether, these findings indicate that NACT not only recruits CD4^+^ T_FH_ cells to the mTME in support of GC formation and B-cell maturation but also promotes the depletion of immunosuppressive TAMs from the metastatic HGSOC microenvironment.

### NACT promotes an ICI-sensitive TCF1^+^PD1^+^CD8^+^ T-cell phenotype in metastatic HGSOC

**Figure 4.**
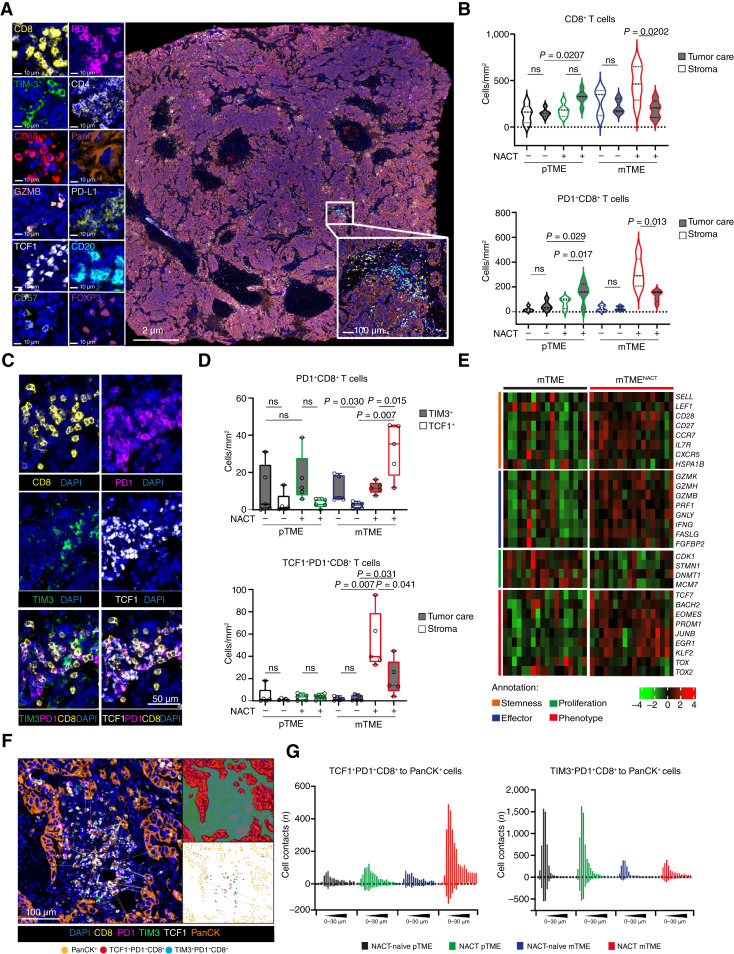
NACT positively increases the ICI-sensitive TCF1^+^PD1^+^CD8^+^ T-cell phenotype within metastatic HGSOC. **A,** Representative image of immunofluorescence of CD68, CD8, PD-L1, FoxP3, TCF1, CD57, PanCK, PD1, CD4, CD20, GZMB, and TIM-3 staining (immunofluorescence panel 2). Scale bars, 2 μm, 10 µm, and 100 µm. **B,** Violin plot showing the density of CD8^+^ and PD1^+^CD8^+^ within tumor core and tumor stroma of pTME and mTME of chemo-naïve and treated HGSOC. Statistical significance was calculated by two-sided Mann–Whitney test. *P* values are indicated. **C** and **D,** Representative image (**C**) and box plot showing the density of TCF1^+^PD1^+^CD8^+^ T cells and TIM-3^+^PD1^+^CD8^+^ and spatial distribution of TCF1^+^PD1^+^CD8^+^ T cells within tumor core and stroma in pTME and mTME of chemo-naïve and treated HGSOC (**D**). Statistical significance was calculated by two-sided Mann–Whitney test. *P* values are indicated. **E,** Supervised hierarchical clustering of gene signatures associated with different stages of T-cell differentiation: T-cell stemness (orange), T-cell effector function (blue), T-cell proliferation (green), T-cell phenotype (red) as determined by RNAseq in mTME of chemo-naïve and treated HGSOC. For further details, see Supplementary Fig. S9. **F** and **G,** Representative image of digital pathology spatial distribution and violin plot showing the number of cell contacts between PanCK^+^ malignant cells and TCF1^+^PD1^+^CD8^+^ T cells and TIM-3^+^PD1^+^CD8^+^ within 0 to 30 µm in pTME and mTME of chemo-naïve and treated HGSOC.

As TLS development has previously been shown to correlate with clonal dominance in both the CD8^+^ and CD4^+^ T-cell compartment and active antitumor immunity (49), we next analyzed the density of CD8^+^ CTLs as a whole as well as CD8^+^ CTLs expressing the activation marker PD1 in tumor nests and the surrounding stroma using immunofluorescence microscopy in HGSOC samples (study cohort 1 and 2; Fig. 4A). Importantly, we observed an increased density of CD8^+^ and PD1^+^CD8^+^ T cells in tumor nests from pTME samples from patients receiving NACT as compared to their NACT-naïve counterparts, as well as compared to mTME samples, in which CD8^+^ and PD1^+^CD8^+^ T cells predominantly localized to the tumor stroma (Fig. 4B).

To obtain additional insights into the PD1^+^CD8^+^ T-cell phenotype elicited by NACT, we differentially analyzed the density of ICI-sensitive TCF1^+^PD1^+^CD8^+^ T cells and ICI-insensitive TIM-3^+^PD1^+^CD8^+^ T cells (12, 14, 50, 51) using immunofluorescence microscopy alongside spatial distribution analyses in 20 pTME and mTME HGSOC samples from study cohort 1 and 2 (Fig. 4C). Digital pathology revealed that TIM-3^+^PD1^+^CD8^+^ T cells accounted for the majority of PD1^+^CD8^+^ T cells within the primary HGSOC microenvironment (irrespective of NACT), as well as within the mTME of patients with NACT-naïve HGSOC (Fig. 4D). Conversely, NACT was associated with a polarization of the PD1^+^CD8^+^ T-cell compartment toward a TCF1^+^PD1^+^ phenotype, especially (but not exclusively) in the tumor stroma (Fig. 4D). These findings were largely confirmed by RNA-seq analyses based on gene signatures associated with T-cell stemness, effector activity, proliferation, and exhaustion/dysfunction (Fig. 4E; Supplementary Fig. S9; ref. 52). Specifically, gene signatures associated with T-cell stemness and effector functions were significantly upregulated in the mTME of patients with HGSOC treated with NACT as compared to their NACT-naïve counterparts (Fig. 4E; Supplementary Fig. S9).

Further digital pathology analyses revealed a significantly increased number of ICI-sensitive TCF1^+^PD1^+^CD8^+^ T cells in close proximity (<10 μm) to PanCyto^+^ tumor cells in the mTME of patients with HGSOC receiving NACT as compared to pTME samples (irrespective of NACT) and mTME samples from NACT-naïve patients (Fig. 4F and G). Conversely, ICI-insensitive TIM-3^+^PD1^+^CD8^+^ T cells were in close proximity (<10 μm) of PanCyto^+^ tumor cells predominanty in pTME samples and mTME biopsies from NACT-naïve patients with HGSOC (Fig. 4G; Supplementary Fig. S10A and S10B).

Altogether, these findings indicate that the mTME of NACT-treated patients with HGSOC exhibit a preferential infiltration by ICI-sensitive, progenitor-like TCF1^+^PD1^+^CD8^+^ CTLs that are localized in the close proximity of malignant cells.

### NACT-associated TCF1^+^PD1^+^CD8^+^ T cells exhibit cytotoxic functions in the metastatic HGSOC microenvironment

**Figure 5.**
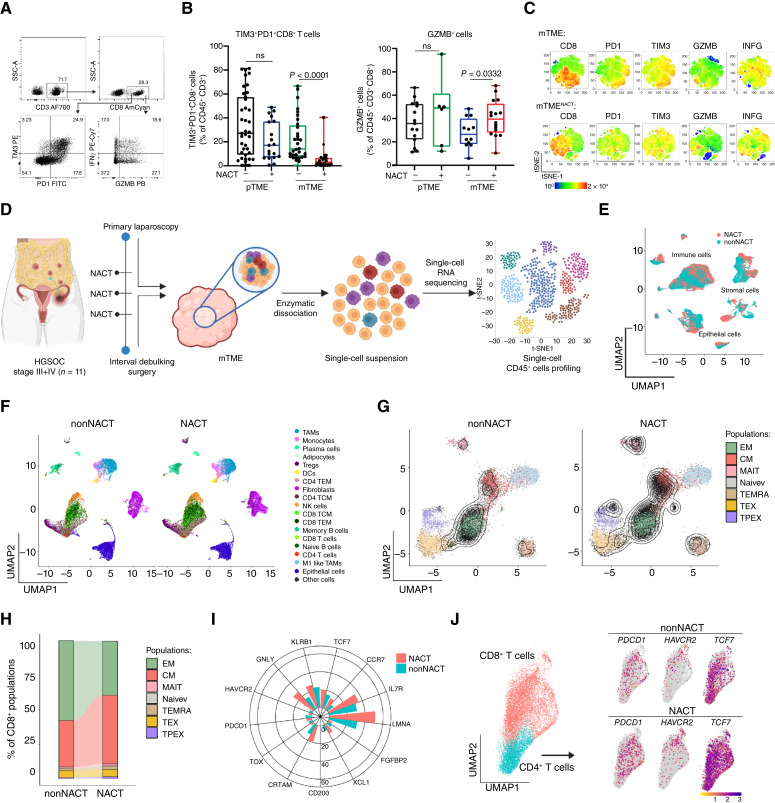
NACT-mediated progenitor TCF1^+^PD1^+^CD8^+^ T-cell phenotype associates with effector cytotoxic functions within metastatic HGSOC. **A–C,** Representative dot plot (**A**) and box plot (**B**) showing percentage of TIM-3^+^PD1^+^CD8^+^ T cells and GZMB^+^CD8^+^ T cells within native pTME and mTME of chemo-naïve and treated HGSOC as determined by flow cytometry. Box plots: lower quartile, median, upper quartile; whiskers, minimum, maximum. Statistical significance was calculated by the Mann–Whitney test. *P* values are indicated. **C, **Marker heatmap dot plots obtained after t-SNE and showing the relative expression of the indicated marker in the different phenotypic clusters within mTME of chemo-naïve and treated HGSOS as determined by flow cytometry. **D,** Design of experimental and sequencing workflow in 11 patients with HGSOC before and after NACT. scRNAseq was performed on dissociated solid tumor specimens using 10× Genomics Chromium platform. **E** and **F,** Uniform manifold approximation and projection (UMAP) plot of all cells (*n* = 51,476) passing the quality control, colored by type of therapy (**E**) and cell type (**F**). **G** and **H,** TILs projections (**G**) and predicted subtype frequencies (**H**) in biopsies from patients with chemo-naïve and treated HGSOC. CM, central memory; EM, effector memory; MAIT, mucosal-associated invariant T cells; PTEX, progenitor exhausted T cells; TEMRA, terminally exhausted T cells; TEX, exhausted T cells. **I** and **J,** Radar plot showing percentage of CD8^+^ T cells expressing respective T-cell marker (*KLRB1*, *TCF7*, *CCR7*, *IL7R*, *LMNA*, *FGFBP2*, *XCL1*, *CD200*, *CRTAM*, *TOX*, *PDCD1*, *HAVCR2*, and *GNLY*; **I**) and UMAP showing expression of *PDCD1*, *HAVCR2* and *TCF7* in CD4^+^ and CD8^+^ T-cell clusters in chemo-naïve and treated HGSOC samples (study cohort 5; **J**), as determined by scRNA-seq. (Panel **D** created with BioRender.com.)

We next decided to validate our digital pathology findings on retrospective patient material (study cohort 1 and 2) with freshly resected primary and metastatic HGSOC samples from two independent prospective cohorts of patients not receiving (study cohort 3) or receiving NACT (study cohort 4; Supplementary Fig. S1; Supplementary Table S2). Supporting our immunostaining data (Fig. 3), the abundance of TIM-3^+^PD1^+^CD8^+^ T cells (as determined by flow cytometry) was significantly reduced in metastatic (but not primary) HGSOC lesions from NACT-treated patients as compared to their NACT-naïve counterparts (Fig. 5A and B). Moreover, mTME samples from patients with HGSOC receiving NACT contained an increased amount of CD8^+^ CTLs staining positively for the cytotoxic effector granzyme B (GZMB) as compared to similar samples from NACT-naïve patients (Fig. 5B), significantly correlating with an increased abundance of IFNG^+^PD1^+^CD8^+^ T cells and a reduced representation of TIM-3^+^PD1^+^CD8^+^ T cells, as demonstrated by unsupervised high-dimensional analyses (Fig. 5C; Supplementary Fig. S11A).

To further validate our findings, we characterized the transcriptional profile of T cells infiltrating HGSOC lesions using prospective HGSOC pairs (*n* = 11) obtained before and after platinum-taxane doublet NACT from a public dataset (Study cohort 5; ref. 37) at the single-cell resolution (Fig. 5D). A total of 51,476 cells, including 16,627 malignant and stromal cells and 34,849 immune cells were processed, leading to the identification of epithelial, stromal, and immune compartments based on graph-based clustering (53) and widely acknowledged markers (Fig. 5E and F; Supplementary Fig. S11B and S11C). In line with our digital pathology and flow cytometry findings, the projection of T cells into a reference atlas (Supplementary Fig. S12A and S12B; ref. 42) demonstrated a decrease in CD8^+^ effector memory T cells and progenitor exhausted T cells, coupled with an increased in central memory T cells in HGSOC samples obtained after NACT (Fig. 5G and H). In addition, CD8^+^ CTLs from post-NACT samples exhibited a significantly higher abundance of several transcripts encoding effector molecules (*GNLY*, *TCF7*, *CCR7*, *XCL1*, *KLRB1*, *IL7R*, and *CRTAM*), while the abundance of *HAVCR2* (which encodes TIM-3) and *TOX* as markers of terminal T-cell exhaustion remained unchanged (Fig. 5I and J).

Taken together, these findings indicate that NACT promotes the accumulation of PD1^+^CD8^+^ T cells into the metastatic HGSOC microenvironment, which preferentially preserve an effector ICI-sensitive TCF1^+^PD1^+^CD8^+^ phenotype.

### A PD1-targeting ICI synergizes with chemotherapy in a syngeneic mouse model of TMB^Hi^ (but not TMB^Lo^) HGSOC

**Figure 6.**
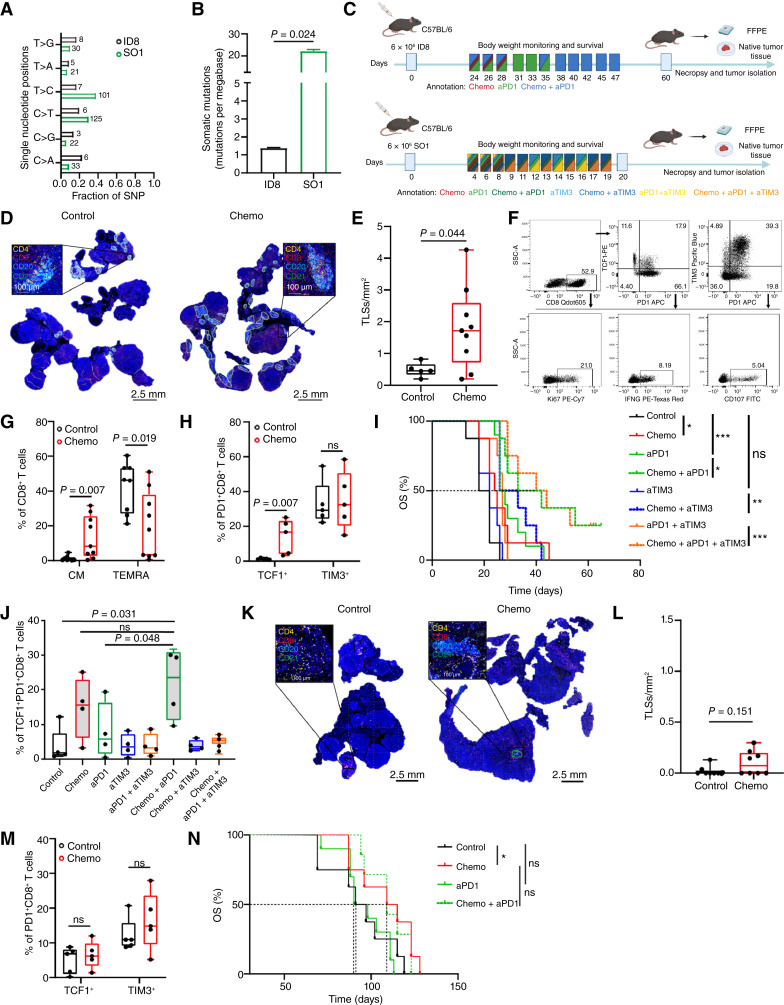
The clinical relevance of combined chemotherapy and immunotherapy in mouse models of TMB^Lo^ and TMB^Hi^ ovarian cancer. **A** and **B,** Bar plots showing single-nucleotide variants positions (SNVs) (**A**) and the somatic mutations prevalence (mutations per megabase) (**B**) in ID8 (*n* = 3) and *Brca1*^−/−^*Trp53*^−/−^/*Myc*/*Hras* SO1 (*n* = 3) C57BL/6 syngeneic mouse ovarian cancer cell lines. Mean and SEM are shown. Statistical significance was calculated by multiple *t* test. *P* values are indicated. **C,** Experimental design for the analysis of TLS aggregates development and efficacy of combined chemotherapy and aPD1 and/or aTIM-3 therapy in TMB^Lo^ ID8 and TMB^Hi^ SO1 experimental syngeneic mouse models. **D** and **E,** Representative immunostaining for CD4, CD8, CD20, and CD21 (**D**) and a box plot showing density of TLS aggregates within chemo-naïve (*n* = 5) and treated TMB^Hi^ SO1 (*n* = 9) ovarian tumors (**E**). Scale bars, 100 µm and 2.5 mm. Box plots: lower quartile, median, upper quartile; whiskers, minimum, maximum. Statistical significance was calculated by two-sided Mann–Whitney test. *P* values are indicated. **F–H,** Representative dot plot (**F**) and flow cytometry analyses for percentages of CD62L^+^CD44^+^ central memory (CM) and CD62L^−^CD44^−^ terminally differentiated CD8^+^ T cells (TEMRA) (**G**) and TCF1^+^PD1^+^CD8^+^ and TIM-3^+^PD1^+^CD8^+^ T cells (**H**) in tumor samples of the TMB^Hi^ SO1 experimental model in the presence or absence of carboplatin and taxane chemotherapy (NACT). Box plots: lower quartile, median, upper quartile; whiskers, minimum, maximum. Statistical significance was calculated by two-sided Mann–Whitney test. *P* values are indicated. **I** and **J,** Overall survival (OS) of TMB^Hi^ SO1 experimental model (**I**) and flow cytometry analyses for percentage of TCF1^+^PD1^+^CD8^+^ T cells after NACT, aPD1, aTIM-3 and combined therapy (**J**). Survival curves were estimated by the Kaplan–Meier method, and differences between groups were evaluated using log-rank test. Box plots: lower quartile, median, upper quartile; whiskers, minimum, maximum. Statistical significance was calculated by two-sided Mann–Whitney test. *P* values are indicated. **K** and **L,** Representative immunostaining for CD4, CD8, CD20, and CD21 (**K**) and a box plot showing density of TLS aggregates within chemo-naïve (*n* = 8) and treated TMB^Lo^ ID8 (*n* = 8) (**L**) ovarian tumors. Scale bars, 100 µm and 2.5 mm. Box plots: lower quartile, median, upper quartile; whiskers, minimum, maximum. Statistical significance was calculated by two-sided Mann–Whitney test. *P* values are indicated. **M,** Flow cytometry analyses for TCF1^+^PD1^+^CD8^+^ and TIM-3^+^PD1^+^CD8^+^ T cells in tumor samples of the TMB^Lo^ ID8 experimental model in the presence or absence of carboplatin and taxane chemotherapy (NACT). Box plots: lower quartile, median, upper quartile; whiskers, minimum, maximum. Statistical significance was calculated by two-sided Mann–Whitney test. *P* values are indicated. **N,** Overall survival (OS) of TMB^Lo^ ID8 experimental model after NACT, anti-PD1, and combined therapy. Survival curves were estimated by the Kaplan–Meier method, and differences between groups were evaluated using log-rank test. *P* values are indicated. (Panel **C** created with BioRender.com.)

To experimentally dissect the potential link between the ability of NACT to alter the immunological configuration of the metastatic HGSOC microenvironment in patients, we harnessed two mouse models of ovarian cancer that exhibit significantly different tumor mutational burden (TMB), namely, ID8 cells and *Brca1*^−*/*−^*Trp53*^−*/*−^*/Myc/Hras* SO1 cells to establish progressive intraperitoneal disease in immunocompetent syngeneic hosts and test the therapeutic efficacy of carboplatin-paclitaxel doublet chemotherapy optionally combined with ICIs (Fig. 6A–C). Immunofluorescence microscopy on TMB^Hi^ SO1 tumors collected after carboplatin-paclitaxel chemotherapy demonstrated an increased number of TLSs as compared to untreated tumors (Fig. 6D and E), with no preferential contribution from carboplatin or paclitaxel when tested individually (Supplementary Fig. S13). These findings were validated by flow cytometry, demonstrating an increase in intratumoral central memory (CD62L^+^CD44^+^) CD8^+^ T cells coupled with a decrease in terminally exhausted (CD62L^−^CD44^−^) CD8^+^ CTLs after chemotherapy (Fig. 6F and G). Of note, although the overall abundance of PD1^+^CD8^+^ T cells infiltrating SO1 tumors remained unchanged after NACT (Supplementary Fig. S14A), they preferentially polarized toward a progenitor-like ICI-sensitive TCF1^+^PD1^+^ phenotype as compared to no changes in the terminally exhausted ICI-insensitive TIM-3^+^PD1^+^ state (Fig. 6H).

Inspired by our preclinical and clinical findings suggesting that NACT polarizes CD8^+^ CTLs infiltrating the metastatic HGSOC microenvironment towards an ICI-sensitive TCF1^+^PD1^+^ phenotype, we experimentally dissected the impact of chemotherapy on therapeutic responses to ICIs targeting PD1 and the terminal exhaustion marker TIM-3 (Fig. 6C). Both chemotherapy (*P* = 0.031) and a PD1 (but not a TIM-3) blocker (*P* = 0.001) employed as standalone therapeutic agents provided a significant survival benefit to C57BL/6mice bearing SO1 lesions (Fig. 6I; Supplementary Fig. S14B). Moreover, chemotherapy combined with PD1 blockage resulted in increased OS as compared to PD1 blockage (*P* = 0.031) and chemotherapy (*P* = 0.015) alone, with virtually no added benefits from the addition of a TIM-3 blockers to both monotherapies or the dual combinatorial regimen (Fig. 6I; Supplementary Fig. S14B). In line with these findings, the relative abundance of TCF1^+^PD1^+^ cells significantly increased in SO1 tumors exposed to carboplatin-paclitaxel, PD1 blockage, and ever more so their combination (Fig. 6J).

Finally, we investigated the sensitivity of progressive TMB^Lo^ ID8 HGSOC lesions established in immunocompetent syngeneic hosts to carboplatin-paclitaxel doublet chemotherapy optionally combined with a PD1-targeting ICI (Fig. 6C). Despite the elevated expression of CALR on the surface of ID8 cells as elicited by carboplatin-paclitaxel (Supplementary Fig. S14C), we failed to observe an increased frequency of TLSs in intraperitoneal ID8 lesions collected after chemotherapy compared to control conditions (Fig. 6K and L). In line with these observations, ID8-infiltrating PD1^+^CD8^+^ T cells preferentially polarized towards a terminally exhausted TIM-3^+^PD1^+^ phenotype that not be altered by chemotherapy (Fig. 6M). Moreover, we failed to observe any positive cooperation between chemotherapy and a PD1 blocker in mice bearing TMB^Lo^ ID8 lesions (Fig. 6N).

Taken together, these findings indicate that in the context of adequate antigenicity, chemotherapy can generate adjuvant-like signals that boost the (spontaneous tendency for the) formation of TLS-like aggregates that support an ICI-sensitive TCF1^+^PD1^+^CD8^+^ T-cell phenotype in mouse models of HGSOC.

## Discussion

At odds with other solid tumors, HGSOC is poorly sensitive to ICIs, at least in part due to a TME that is poorly permissive for anticancer immunity at baseline, at least based on primary tumor biopsies (13). Here, we harnessed five independent cohorts of patients with HGSOC to demonstrate that platinum-taxane doublet NACT is associated not only with the emission of adjuvant-like signals such as the exposure of CALR on the surface of malignant cells but also with the establishment of an immunologically permissive metastatic (but not primary) TME associated with increased densities of CD8^+^ T cells, PD1^+^CD8^+^ T cells, CD20^+^ B cells, CD4^+^CXCR5^+^PD1^+^FOXP3^−^ T_FH_ cells, and decreased density of immunosuppressive TAMs, which is in line with previous observations from us and others (29, 32, 54–58). Moreover, we documented a positive impact of NACT on frequency and the level of maturation of TLSs along with increased level of TLS-driving chemokines (*CCL2*, *CLL3*, *CCL4*, *CCL5*, *CCL18*, *CCL19*, *CXCL9*, and *CXCL13*) and a predominance of ICI-sensitive TCF1^+^PD1^+^CD8^+^ CTLs at metastatic (but not primary) HGSOC sites, corroborating the notion that TLSs contribute to the development of anticancer immunity *in situ* (12, 48, 59, 60). These findings are important as they suggest that the immunological configuration of the metastatic HGSOC microenvironment, but less so its primary counterpart (which is most often used for pathology and decision making in the clinic), may at least partially inform on ICI sensitivity after NACT. The precise reasons underlying the differential response of the primary versus metastatic TME to NACT have not yet been clarified but may relate to histological and/or environmental conditions (61, 62).

We also demonstrated that TIM-3^+^PD1^+^CD8^+^ T cells account for the majority of PD1^+^CD8^+^ T cells within primary and chemo-naïve metastatic HGSOC and predominantly localized within tumor cores. In this setting, the NACT-mediated upregulation of TCF1^+^PD1^+^CD8^+^ T cells as observed in both patients with HGSOC and syngeneic preclinical HGSOC models, provided a clear ground for aPD1 blockers to positively interact with chemotherapy in the latter setting. However, the same did not hold true for a TIM-3 blocker, as demonstrated in both TMB^Lo^ and TMB^Hi^ HGSCO models (63). This align with previous findings from us and others demonstrating a key role for TIM-3 in the establishment of local immunosuppression in primary HGSOC and poor response to ICIs targeting PD1 signaling (38, 63, 64).

These data from two syngeneic HGSOC models differing from each other with respect to TMB and propensity of generate TLSs lent additional support to the notion that in the context of sufficient antigenicity, the ability of chemotherapy to promote TLS formation can be harnessed to generate a TME that responds to ICIs. One caveat of these latter findings relates to the use of two distinct HGSOC cell lines, one of which (SO1 cells) exhibits a considerably more aggressive behavior than the other (ID8 cells), despite a superior capacity to establish TLSs. Why the natural formation of TLSs in progressing SO1 tumors is insufficient to delay disease progression but enables synergy between chemotherapy and PD1 blockage is a matter of ongoing investigation.

An unavoidable caveat of NACT studies is related to post-NACT samples collection, when patients have typically received half the number of chemotherapy cycles they will ultimately receive. Thus, NACT-mediated immune patterns might undergo further changes during subsequent cycles of chemotherapy. At least in part addressing this limitation, our study focused on large retrospective and prospective sample cohorts encompassing paired primary and metastatic HGSOCs tissue from NACT-naïve and NACT-treated patients that were collected 21 days after no less than three cycles of NACT (study cohorts 1–4). While this excluded from our study early complete responders to NACT, as their samples contained insufficient material for analysis, we confirmed our findings on paired treatment-naïve and treated HGSOC samples collected after three cycles of platinum-taxane NACT at the single-cell resolution (study cohort 5).

Both paclitaxel and carboplatin have been previously associated with immunostimulatory effects resulting from (among other pathways) ER stress coupled with polyploidization (28, 65), SNARE-dependent vesicle exocytosis (55), and increased expression of MHC Class I molecules on the cell surface (66). Supporting this notion, ER stress-related chaperones other than CALR including heat shock protein family A (Hsp70) member 1A (HSPA1A, best known as HSP70) and heat shock protein 90 alpha family class A member 1 (HSP90AA1, best known as HSP90) were also upregulated by NACT in the mTME of patients with HGSOC. Of note, in a preclinical mouse models of TMB^Hi^ HGSOC, both platinum and carboplatin applied as monotherapy promoted similar numbers of TLSs.

At least in some clinical indications, first-line chemotherapy appears to positively cooperate with immunotherapy (57, 67, 68). Our findings align with these observations at they suggest that rationally designed treatment combinations and optimal administration schedules are critical to circumvent immunosuppression in the HGSOC microenvironment and hence restore clinical sensitivity to ICIs. Moreover, our findings indicate that the metastatic HGSOC microenvironment, and notably its enrichment for TLSs, may offer a preferential platform to identify patients who after NACT and surgical tumor removal may benefit from ICIs targeting PD1. Additional clinical work is required to formally dissect this possibility.

## Supplementary Material

Supplementary Data1 Supplemental material, figures and tables
