# Supplementary material for: Chemotherapy Drives Tertiary Lymphoid Structures That Correlate with ICI-Responsive TCF1+CD8+ T Cells in Metastatic Ovarian Cancer
Source: Clin Cancer Res. 2024 Aug 20;31(1):164–80. doi: 10.1158/1078-0432.CCR-24-1594 (PMC11701433; doi:10.1158/1078-0432.CCR-24-1594)
Supplement: Supplementary Data1 — Supplemental material, figures and tables [file ccr-24-1594_supplementary_data1_suppds1.pdf]

Supplemental Materials and Methods

DNA/RNA isolation from FFPE. RNA and DNA were isolated using AllPrep DNA/RNA FFPE Kit (Qiagen) according to manufacturer instructions. RNA concentration and purity were determined using a NanoDrop 2000c (Thermo Scientific, Germany). Samples were stored at −80°C until further use.

Bulk RNA sequencing and analysis. Raw FASTQ sequencing files were aligned to human reference genome (build h19) with bowtie2 (version 2.3.2) and tophat2 (version 2.1). Expression levels as raw "counts" were calculated from aligned reads with mapping quality ≥10 using htseq-count (version 0.6.0). Differential gene expression analyses were performed using DESeq2 (version 1.24.0) in R. Unsupervised hierarchical clustering heatmaps were used for differentially expressed genes (DEGs) using the R package ComplexHeatmap (2.8.0) based on the Euclidean distance and Ward2 clustering method. R package ggplot2 (3.3.6) was used for alluvial and stacked plot.

Sequential immunohistochemistry (IHC) protocol for detection follicular T cells. Immunostaining with antibodies specific for CXCR5, programmed cell death 1 (PDCD1, best known as PD1), FoxP3, CD23, CD20, and CD4 was performed according to sequential protocol. Briefly, tumor specimens were fixed in neutral buffered 10% formalin solution and embedded in paraffin as per standard procedures. In brief, 4 µm-thick tissue sections were deparaffinized and rehydrated in a descending alcohol series (100, 96, 70, and 50%), followed by antigen retrieval with Target Retrieval Solution (Leica) in pH 9 (for first sequential staining antibody CXCR5) in a preheated water bath (97°C, 30 min). Sections were allowed to cool down to RT for 30 min. Endogenous peroxidase or alkaline phosphatase activity was blocked with blocking solution Bloxall (Vector), for 10 minutes. Thereafter, sections were treated with Normal Horse Serum 2,5% (Vector) for 20 min and incubated with anti-CXCR5 primary antibodies (1:500, 60 min) (**Suppl. Table 3**), followed by the revelation of enzymatic activity (AEC substrate, Vector). Sections were counterstained with hematoxylin (DAKO) for 2 mins. Images were acquired using a Leica Aperio AT2 scanner (Leica). Thereafter the same sections were decolorated and antibody was removed by heating in pH9 in microwave for 30 min. Sections were allowed to cool down to RT for 30 min. Endogenous peroxidase or alkaline phosphatase activity was blocked with blocking solution Bloxall (Vector), for 10 minutes. Thereafter, sections were treated with Animal free blocker and Diulent R.T.U (Vector) for 60 min and incubated with anti-PD1 primary antibodies (1:50,

120 min) (**Suppl. Table 3**), followed by the revelation of enzymatic activity (AEC substrate, Vector). Sections were counterstained with hematoxylin (DAKO) for 2 mins. Images were acquired using a Leica Aperio AT2 scanner (Leica). The same sections were decolorated and antibody was removed by heating in pH6 in water bath 97°C for 30 min. Section were allowed to cool down to RT for 30 min. Endogenous peroxidase or alkaline phosphatase activity was blocked with blocking solution Bloxall (Vector), for 10 minutes. Thereafter, sections were treated with Normal Horse Serum 2,5% (Vector) for 20 min and incubated with anti-FoxP3 primary antibodies (1:50, 120 min) (**Suppl. Table 3**), followed by the revelation of enzymatic activity (AEC substrate, Vector). Sections were counterstained with hematoxylin (DAKO) for 2 mins. Images were acquired using a Leica Aperio AT2 scanner (Leica). The same sections were decolorated and antibody was removed by heating in pH8 in microwave for 30 min. Section were allowed to cool down to RT for 30 min. Endogenous peroxidase or alkaline phosphatase activity was blocked with blocking solution Bloxall (Vector), for 10 minutes. Thereafter, sections were treated with Normal Horse Serum 2,5% (Vector) for 20 min and incubated with anti-CD23 primary antibodies (1:200, 60 min) (**Suppl. Table 3**), followed by the revelation of enzymatic activity (AEC substrate, Vector). Sections were counterstained with hematoxylin (DAKO) for 2 mins. Images were acquired using a Leica Aperio AT2 scanner (Leica). The same sections were decolorated and antibody was removed by heating in pH9 in microwave for 30 min. Section were allowed to cool down to RT for 30 min. Endogenous peroxidase or alkaline phosphatase activity was blocked with blocking solution Bloxall (Vector), for 10 minutes. Thereafter, sections were treated with Normal Horse Serum 2,5% (Vector) for 20 min and incubated with anti-CD20 primary antibodies (1:50, 60 min) (**Suppl. Table 3**), followed by the revelation of enzymatic activity (AEC substrate, Vector). Sections were counterstained with hematoxylin (DAKO) for 2 mins. Images were acquired using a Leica Aperio AT2 scanner (Leica). The same sections were decolorated and antibody was removed by heating in pH8 in microwave for 30 min. Section were allowed to cool down to RT for 30 min. Endogenous peroxidase or alkaline phosphatase activity was blocked with blocking solution Bloxall (Vector), for 10 minutes. Thereafter, sections were treated with Normal Horse Serum 2,5% (Vector) for 20 min and incubated with anti-CD4 primary antibodies (1:50, 120 min) (**Suppl. Table 3**), followed by the revelation of enzymatic activity (DAB substrate, DAKO). Sections were counterstained with hematoxylin (DAKO) for 2 mins. Final image was composed from individual staining sequential steps by the HALO10 software (Indica labs) using the deconvolution and registration algorithm.

Supplemental Figures

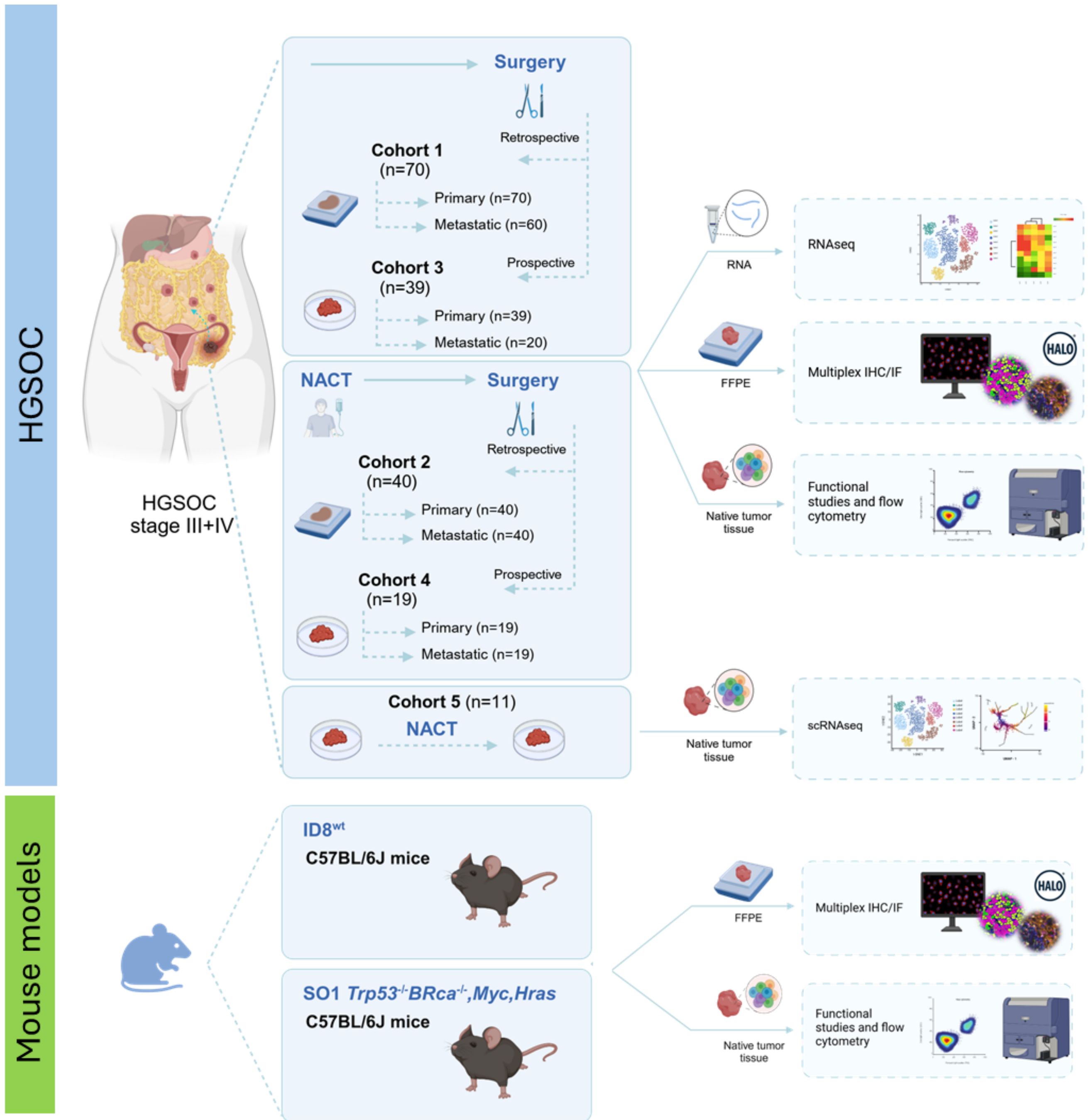

***Supplemental Figure 1.* Experimental design of the study.** Created with BioRender.com.

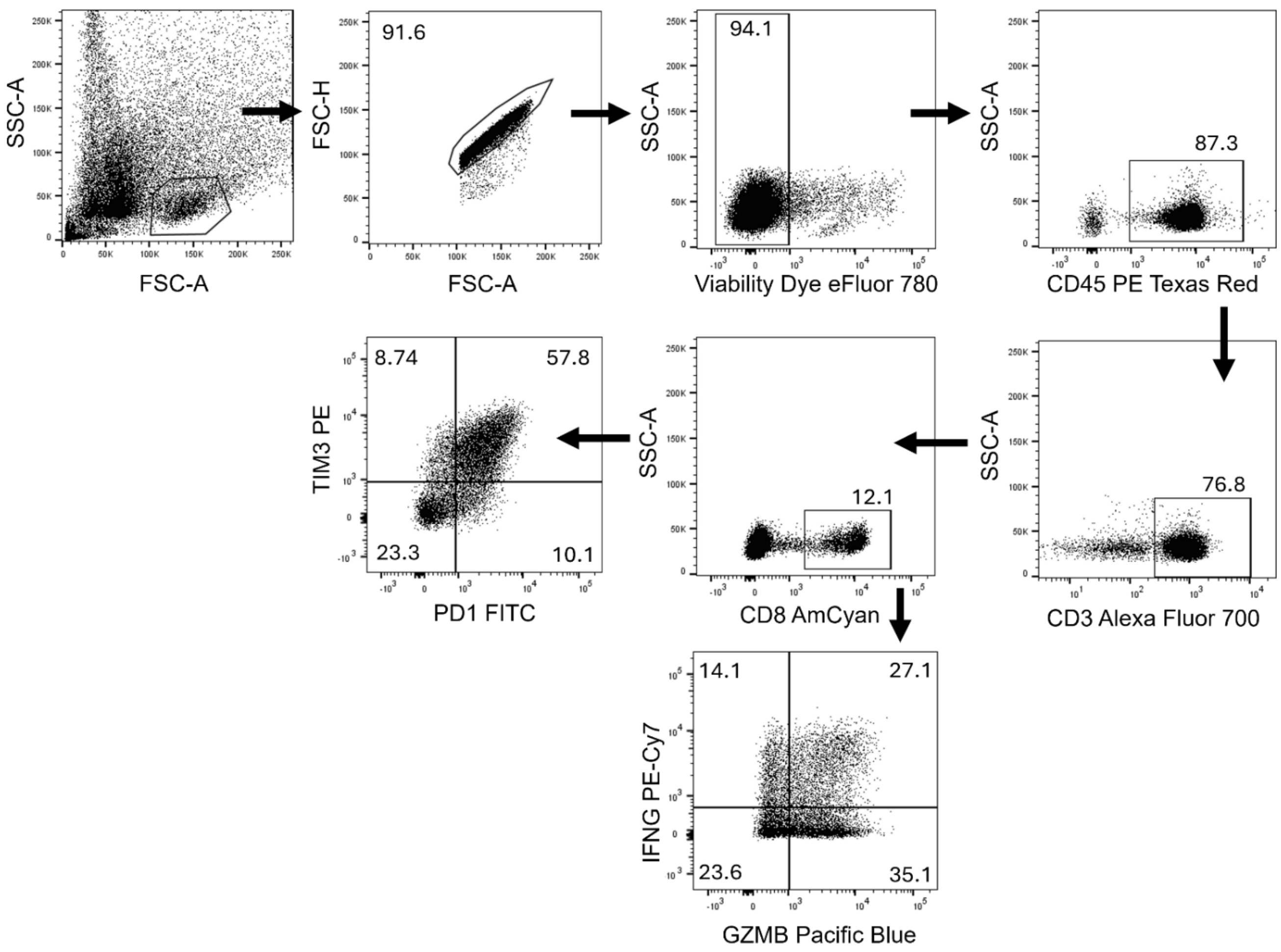

Supplemental Figure 2. **Gating strategy.** Detection of relative numbers of $PD1^+TIM3^+CD8^+$ T cells and $GZMB^+$ $CD8^+$ T cells and $IFNG^+$ $CD8^+$ T cells. The percentage of cells in each gate is reported.

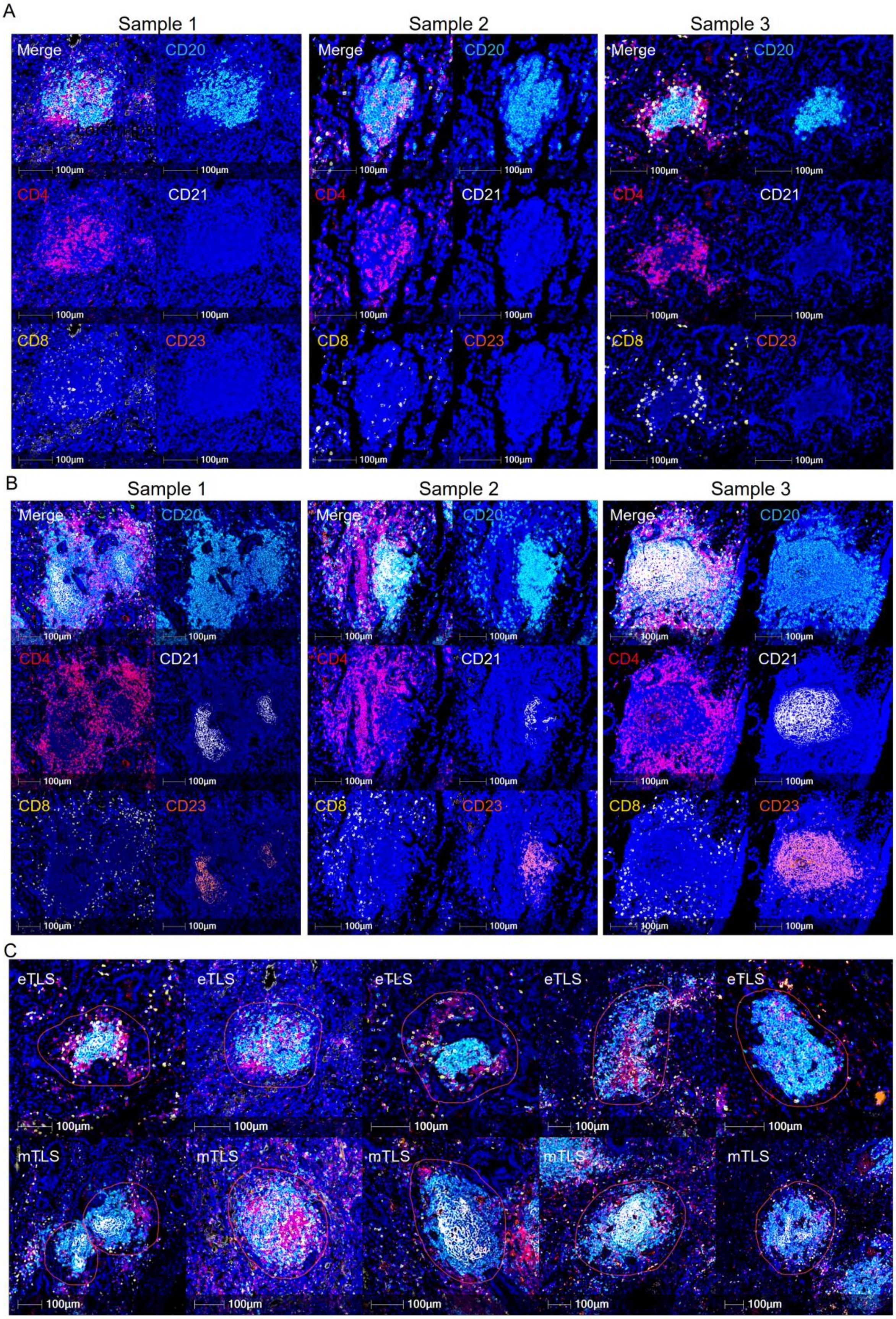

Supplemental Figure 3. **Spatial distribution of eTLS and mTLS. (A, B)** Representative images of eTLS **(A)** and mTLS **(B)** using immunofluorescence of CD4, CD8, CD20, CD21 and CD23 staining. **(C)** Representative image of TLS areas quantification in HALO software. Scale bar 100 μm.

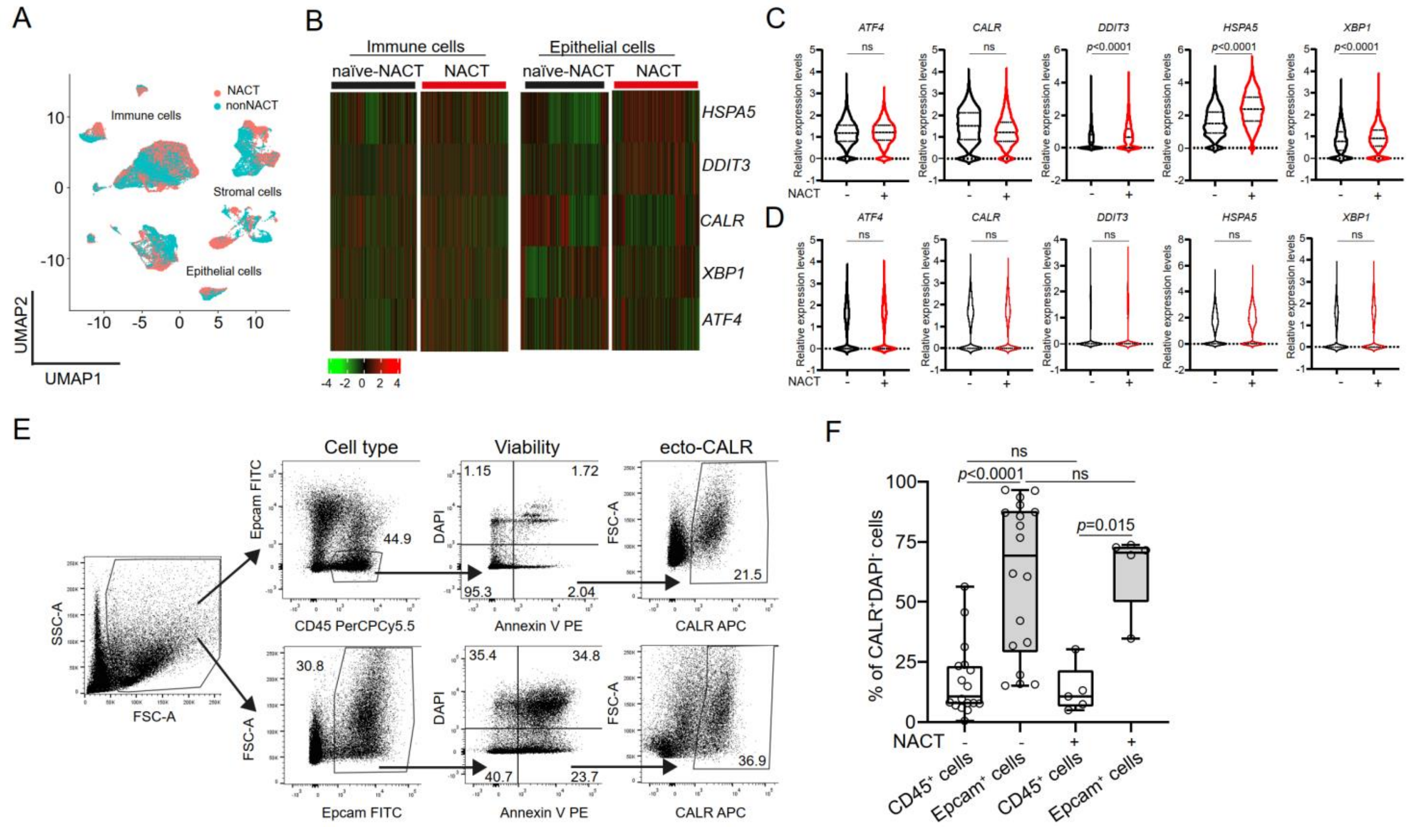

Supplemental Figure 4. **Impact of NACT on ecto-CALR expression in immune cells and malignant cells of HGSOC. (A)** Uniform manifold approximation and projection (UMAP) plot of all cells (n=51.476) passing the quality control, coloured by type of therapy and cell type in biopsies from chemo-naïve and treated HGSOC patients (study cohort 5 of our study). **(B, C)** Supervised heatmap and violin plot showing expression of ER stress associated genes: *ATF4, CALR, DDIT3, HSPA5* and *XBP1* in chemo-naïve and treated samples of HGSOC patients, determined by scRNAseq. Statistical significance was calculated by the Mann–Whitney test. *p* values are indicated. **(E, F)** Representative dot plot **(E)** and box plot **(F)** showing percentage of ecto-CALR on CD45^{+}DAPI^{-} immune cells and Epcam^{+}DAPI^{-} tumor cells within native pTME of chemo naïve (study cohort 3) and treated (study cohort 4) HGSOC as determined by flow cytometry. Box plots: lower quartile, median, upper quartile; whiskers, minimum, maximum. Statistical significance was calculated by the Mann–Whitney test. *p* values are indicated.

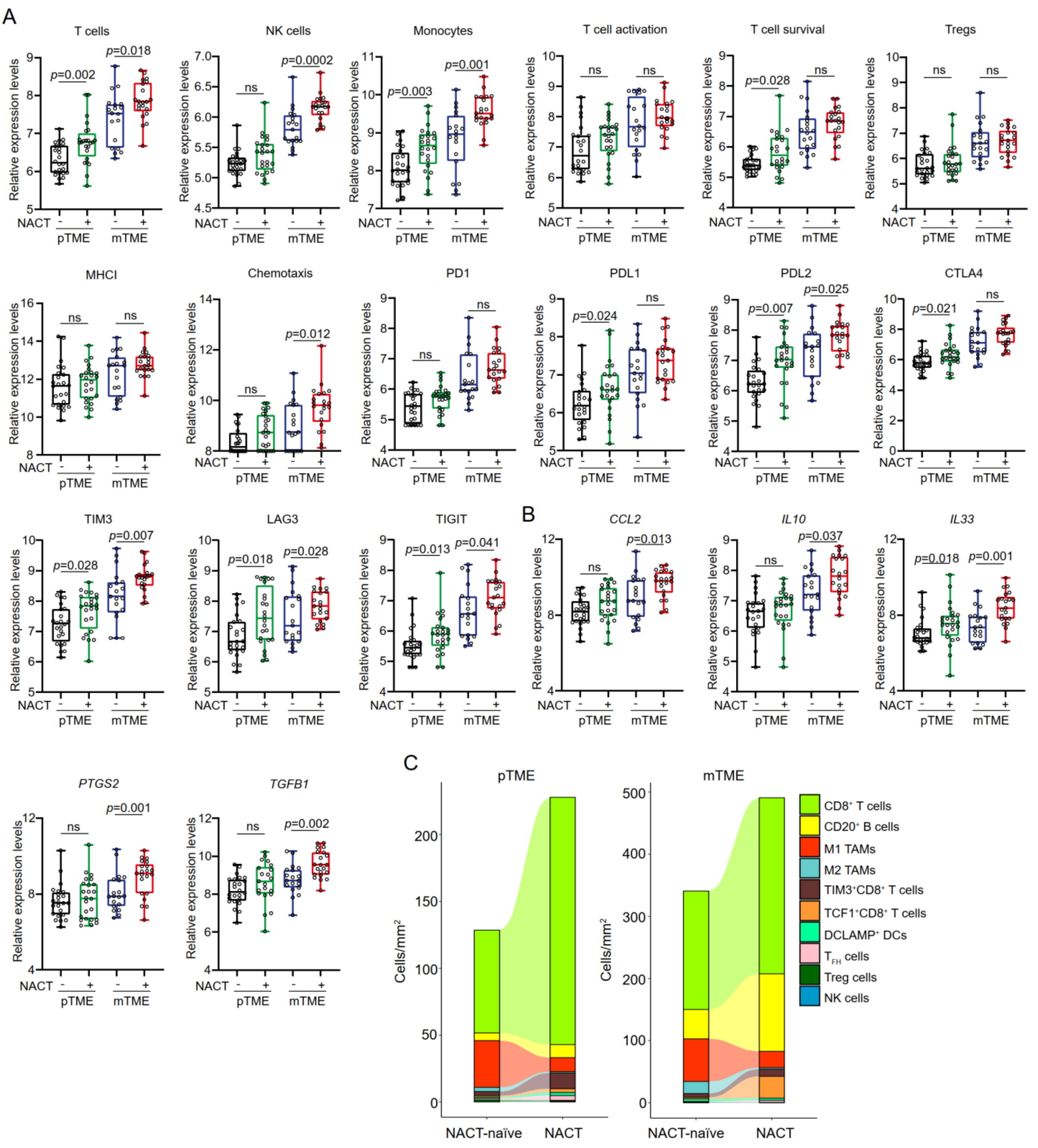

Supplemental Figure 5. **Immunomodulation by neo-adjuvant chemotherapy (NACT) in metastatic HGSOC. (A, B)** Gene expression signature associated with T cells, NK cells, monocytes, T cell activation, T cell survival, Tregs, MHCI, chemotaxis, TLS, PD1, PDL1, PDL2, CTLA4, TIM3, LAG3, TIGIT **(A)** and immunosuppressive cytokines (*CCL2*, *IL10*, *IL33*, *PTGS2* and *TGFB1*) **(B)** as determined on RNAseq data from pTME and mTME HGSOC with/without NACT. Box plots: lower quartile, median, upper quartile; whiskers, minimum, maximum. Statistical significance was calculated by two-sided the Mann–Whitney test.

p values are indicated. Abbreviations: IS, immunosuppression; TLS, tertiary lymphoid structures. **(C)** Stacked plot representing median density of $CD8^+$ T cells, $CD20^+$ B cells, M1 like TAMs, M2 like TAMs, $TIM3^+PD1^+CD8^+$ T cells, $TCF1^+PD1^+CD8^+$ T cells, DC-LAMP$^+$ cells, T_{FH} cells, Treg cells and NK cells as determined by immunostaining in pTME and mTME (Study cohort 1) HGSOC samples with/without NACT.

A

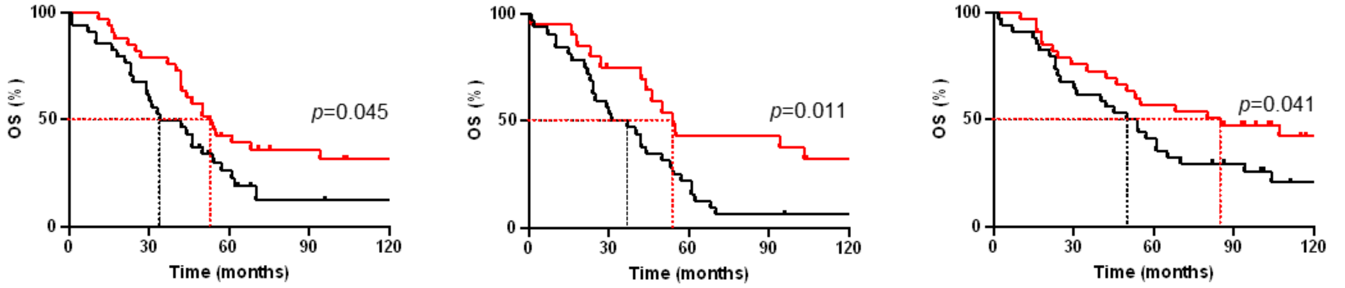

Time	0	30	60	90	120
DC-LAMP^{lo}	34	20	8	3	2
DC-LAMP^{hi}	33	27	14	9	6

Time	0	30	60	90	120
CD20^{lo}	32	19	8	3	2
CD20^{hi}	20	15	9	9	6

Time	0	30	60	90	120
CD8^{lo}	36	23	15	9	4
CD8^{hi}	34	26	19	15	7

B

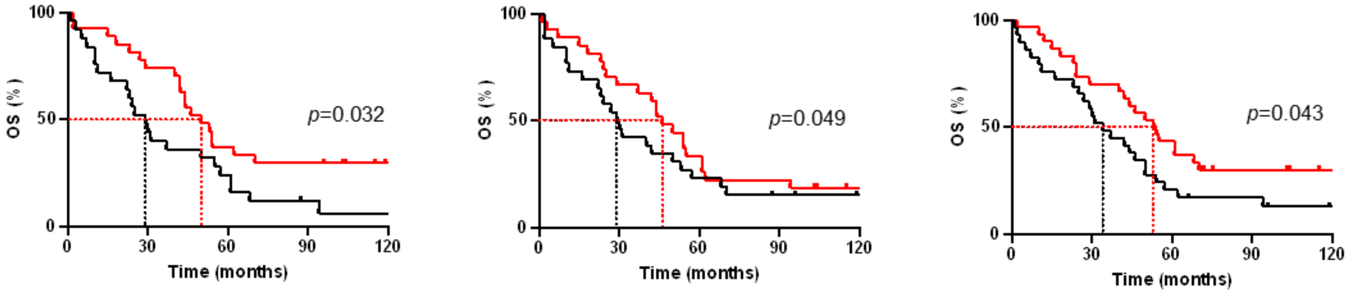

Time	0	30	60	90	120
DC-LAMP^{lo}	25	13	7	3	2
DC-LAMP^{hi}	27	21	12	9	4

Time	0	30	60	90	120
CD20^{lo}	26	14	7	4	2
CD20^{hi}	27	19	10	7	3

Time	0	30	60	90	120
CD8^{lo}	30	18	7	5	2
CD8^{hi}	30	22	14	7	4

C

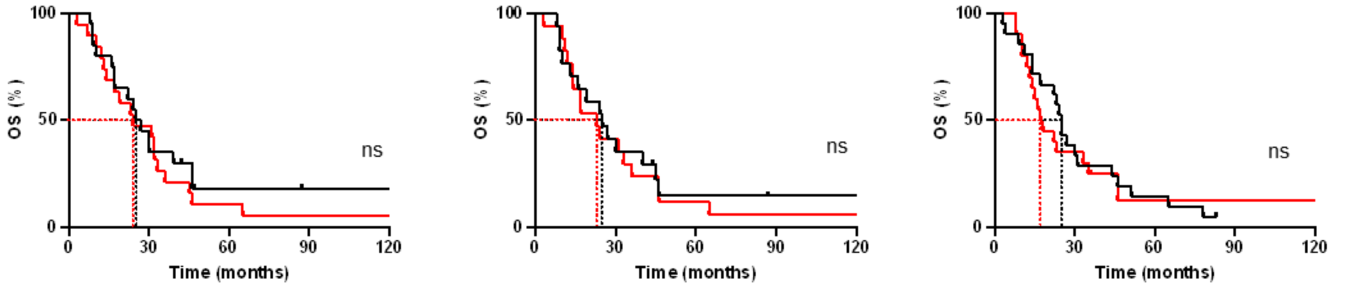

Time	0	30	60	90	120
DC-LAMP^{lo}	20	10	3	2	2
DC-LAMP^{hi}	20	10	3	2	2

Time	0	30	60	90	120
CD20^{lo}	20	8	3	2	2
CD20^{hi}	20	8	4	2	2

Time	0	30	60	90	120
CD8^{lo}	20	7	2	2	2
CD8^{hi}	20	13	4	2	2

D

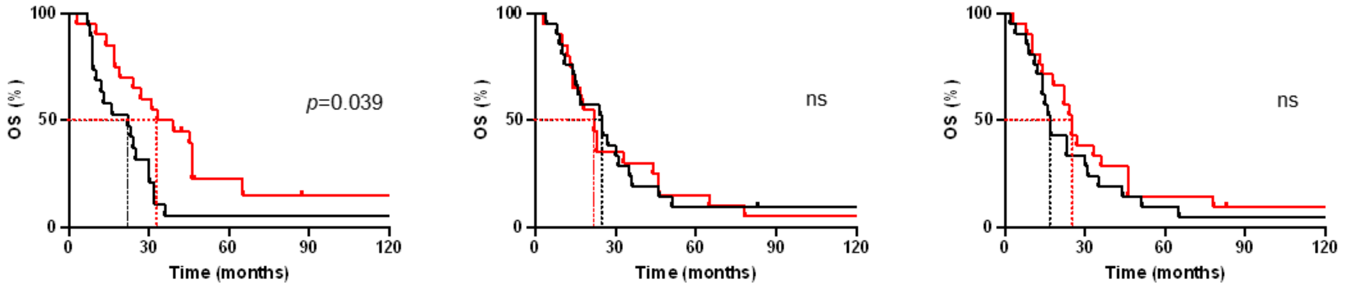

Time	0	30	60	90	120
DC-LAMP^{lo}	20	9	4	1	1
DC-LAMP^{hi}	20	8	4	4	4

Time	0	30	60	90	120
CD20^{lo}	20	9	3	2	2
CD20^{hi}	20	9	5	2	2

Time	0	30	60	90	120
CD8^{lo}	20	9	3	2	2
CD8^{hi}	20	9	6	2	2

Supplemental Figure 6. **The clinical relevance of DC-LAMP$^+$ DCs, CD20$^+$ B cells and CD8$^+$ T cells within pTME and mTME of chemo naïve (study cohort 1) and chemo treated (study cohort 2) HGSOC patients. (A, B)** Overall survival (OS) of 70 HGSOC patients (Study cohort 1) who underwent primary surgery in the absence of neo-adjuvant chemotherapy based on median stratification of density of DC-LAMP$^+$, CD20$^+$ B cells and CD8$^+$ T cells in pTME (A) and mTME (B). **(C, D)** Overall survival (OS) of 40 HGSOC patients who underwent primary surgery after 3 cycles of neo-adjuvant paclitaxel-carboplatin based on median stratification of density of DC-LAMP$^+$, CD20$^+$ B cells and CD8$^+$ T cells in pTME (C) and mTME (D). Survival curves were estimated by the Kaplan-Meier method, and differences between groups were evaluated using log-rank test. Number of patients at risk and *p* values are reported.

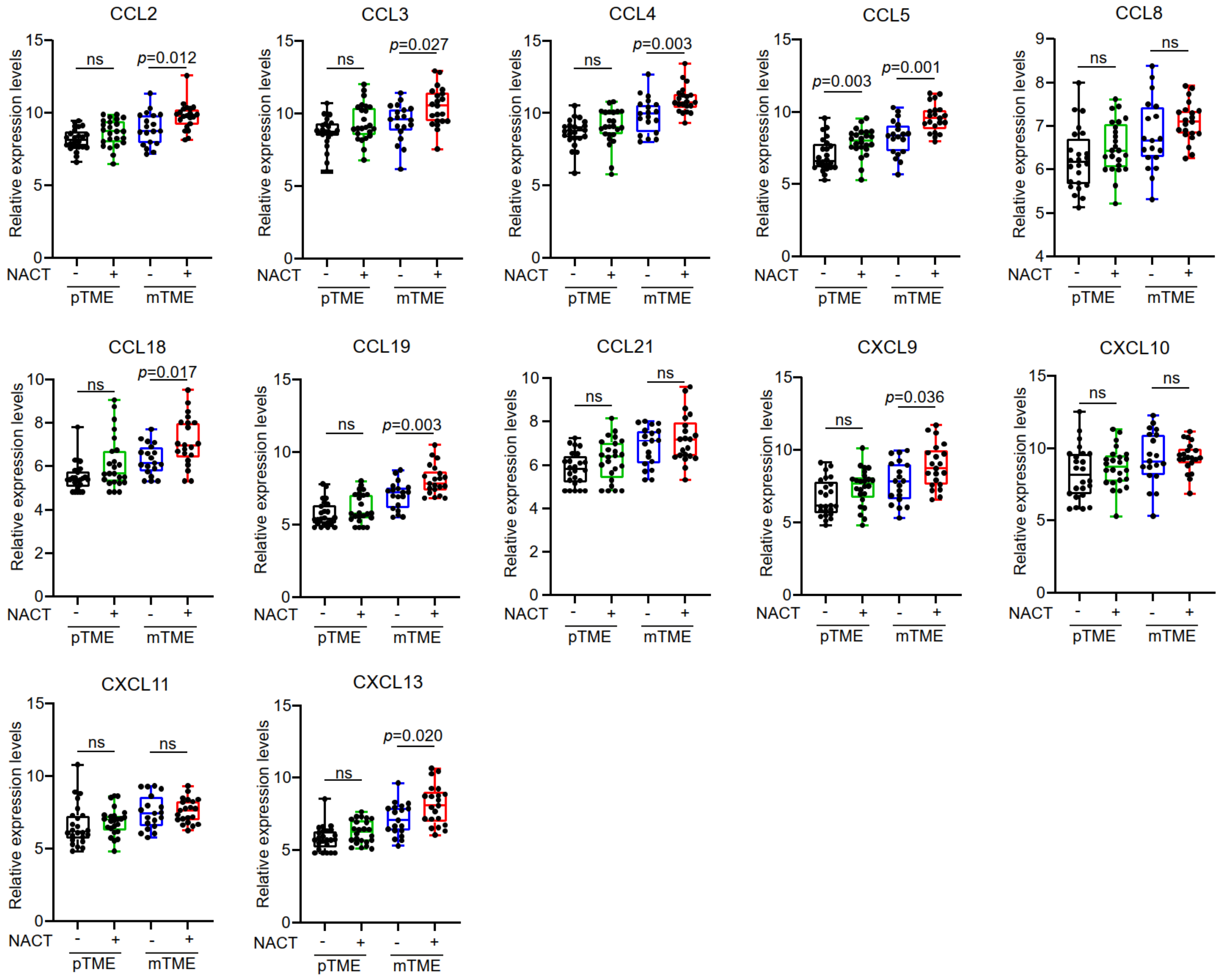

Supplemental Figure 7. **NACT positively impact TLS formation and maturation in metastatic HGSOC.** **(A)** Gene expression of TLS-like genes: *CCL2*, *CCL3*, *CCL4*, *CCL5*, *CCL8*, *CCL18*, *CCL19*, *CCL21*, *CXCL9*, *CXCL10*, *CXCL11*, and *CXCL13* across pTME and mTME HGSOC tumor samples with/without NACT as determined by RNAseq (Study cohort 1 and 2). Box plots: lower quartile, median, upper quartile; whiskers, minimum, maximum. Statistical significance was calculated by two-sided the Mann–Whitney test. *p* values are indicated.

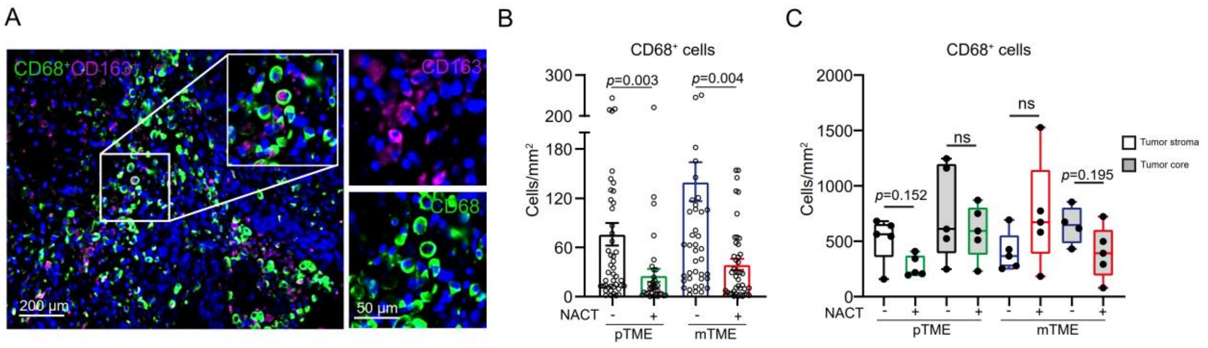

Supplemental Figure 8. **The impact of NACT on density of CD68$^+$ TAMs in pTME and mTME HGSOC. (A, B)** Representative image (A) and density of CD68$^+$ TAMs in pTME and mTME of chemo naïve and treated HGSOC (B). Mean and SEM are shown. Statistical significance was calculated by two-sided Mann-Whitney test. *p* values are indicated. **(C)** Box plot representing density of CD68$^+$ TAMs within tumor stroma and tumor core in pTME and mTME of chemo naïve and treated HGSOC. Box plots: lower quartile, median, upper quartile; whiskers, minimum, maximum. Statistical significance was calculated by the Mann–Whitney test. *p* values are indicated.

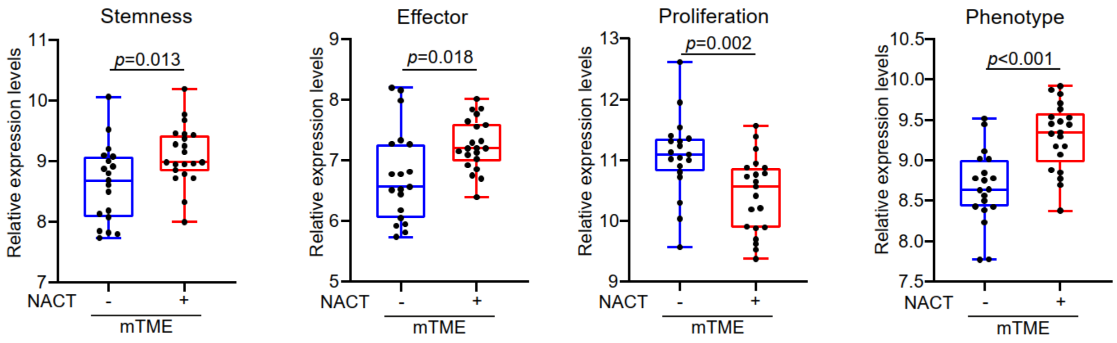

Supplemental Figure 9. **NACT positively impact CD8$^+$ T cells phenotype in metastatic HGSOC.** Gene expression of T cells differentiation gene signatures: T cell stemness (*SELL, LEF1, CD28, CD27, CCR7, IL7R, CXCR5, HSPA1B*), T cell effector functions (*GZMK, GZMH, GZMB, PRF1, GNLY, IFNG, FASLG, FGFBP2*), T cell proliferation (*CK1, STMN1, DNMT1, MCM7*), T cell phenotype *(TCF7, EOMES, PRDM1, JUNB, EGR1, KLF2, TOX, TOX2*) in chemo-naïve and treated mTME of HGSOCs (Study cohort 1, 2) as determined by RNAseq. Box plots: lower quartile, median, upper quartile; whiskers, minimum, maximum. Statistical significance was calculated by two-sided the Mann–Whitney test. *p* values are indicated.

A

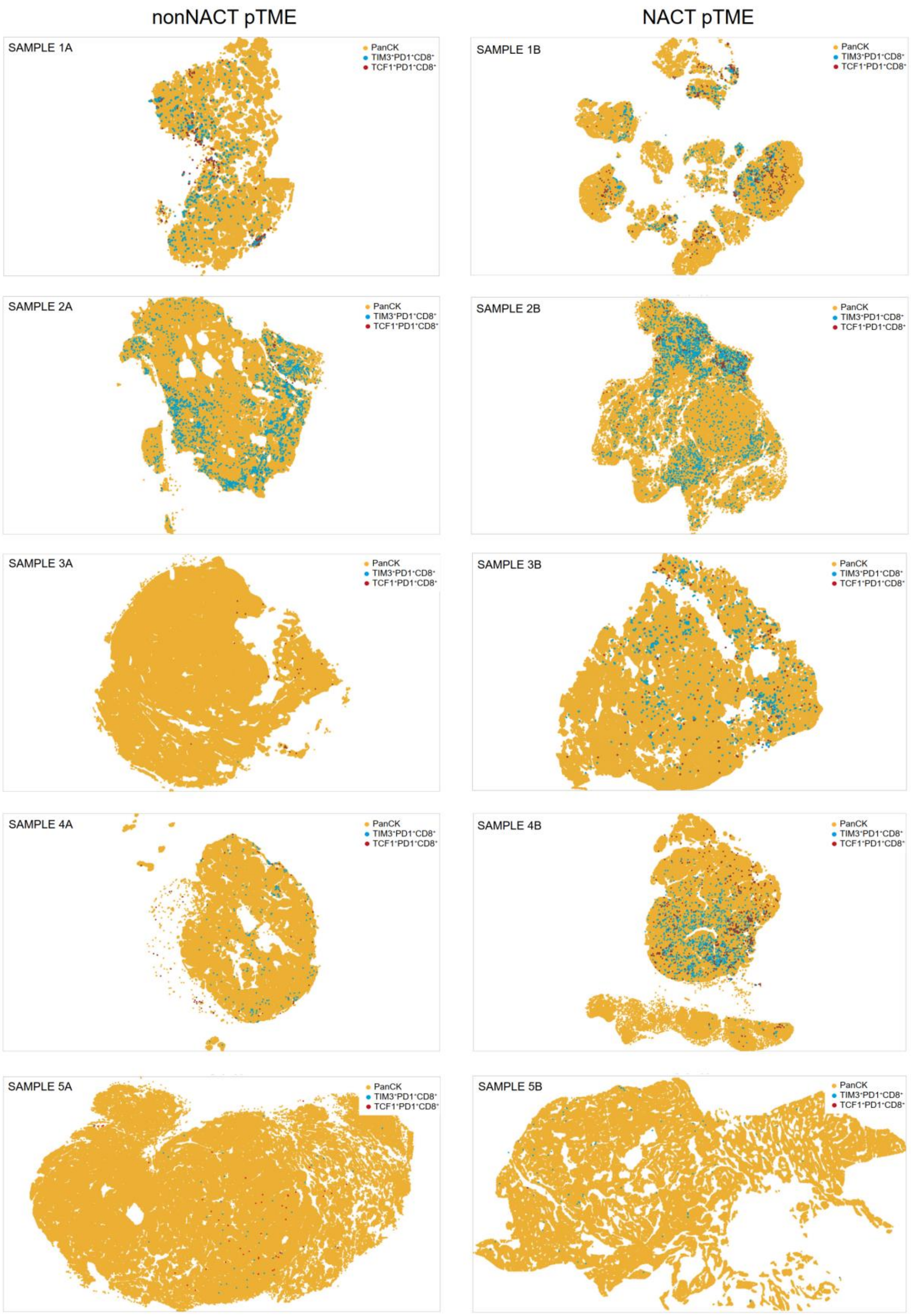

nonNACT pTME
NACT pTME
SAMPLE 1A
SAMPLE 1B
SAMPLE 2A
SAMPLE 2B
SAMPLE 3A
SAMPLE 3B
SAMPLE 4A
SAMPLE 4B
SAMPLE 5A
SAMPLE 5B
PanCK
TIM3+PD1+CD8+
TCF1+PD1+CD8+

B

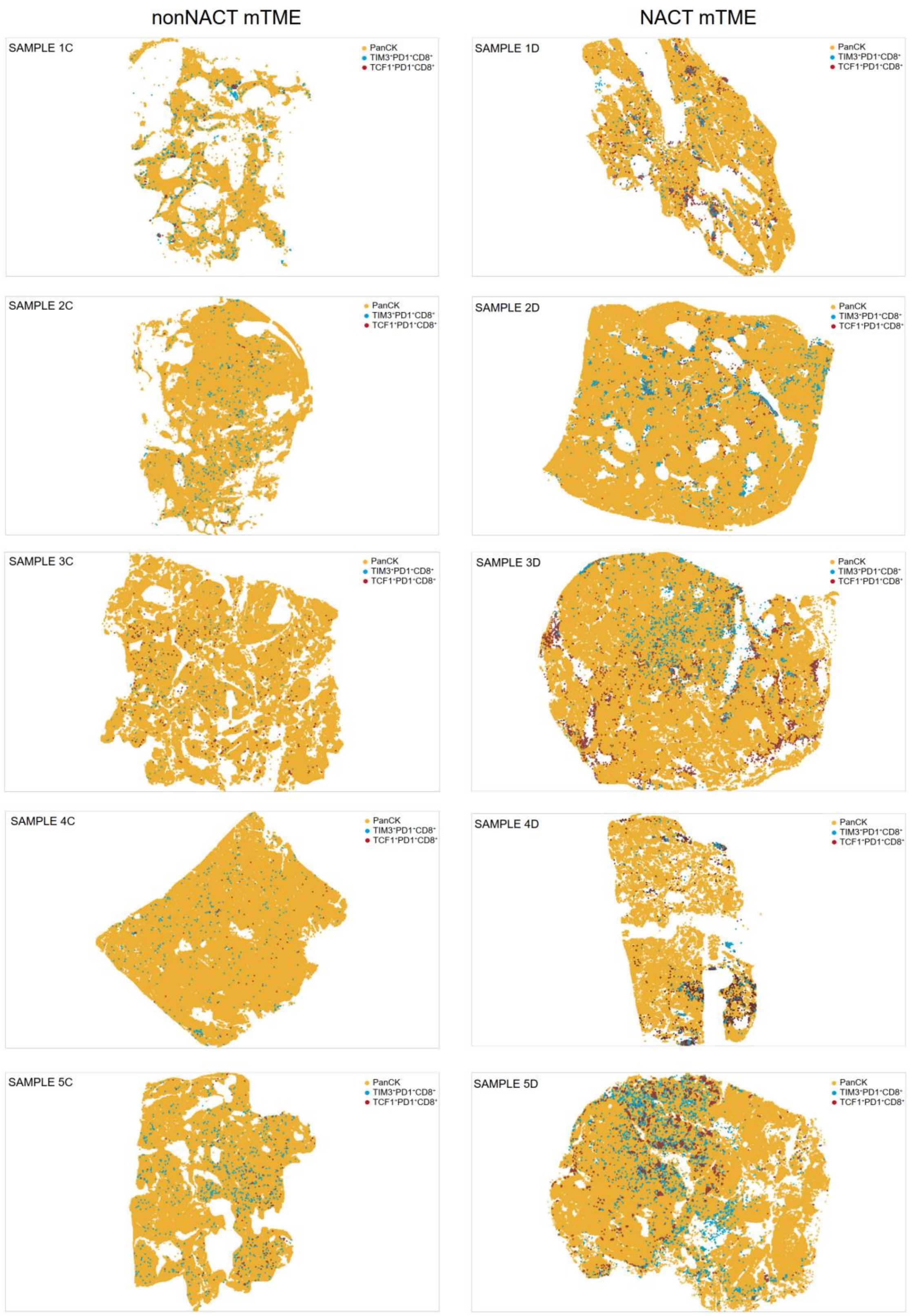

Supplemental Figure 10. **Spatial distribution of TCF1^{+}PD1^{+}CD8^{+} T cells and TIM3^{+}PD1^{+}CD8^{+} T cells within primary and metastatic HGSOC. (A, B)** Representative images of digital pathology spatial distribution analyses of TCF1^{+}PD1^{+}CD8^{+} T cells and TIM3^{+}PD1^{+}CD8^{+} T cells in pTME (A) and mTME (B) of chemo-naïve and chemo-treated samples using HALO10 software (Indica Labs).

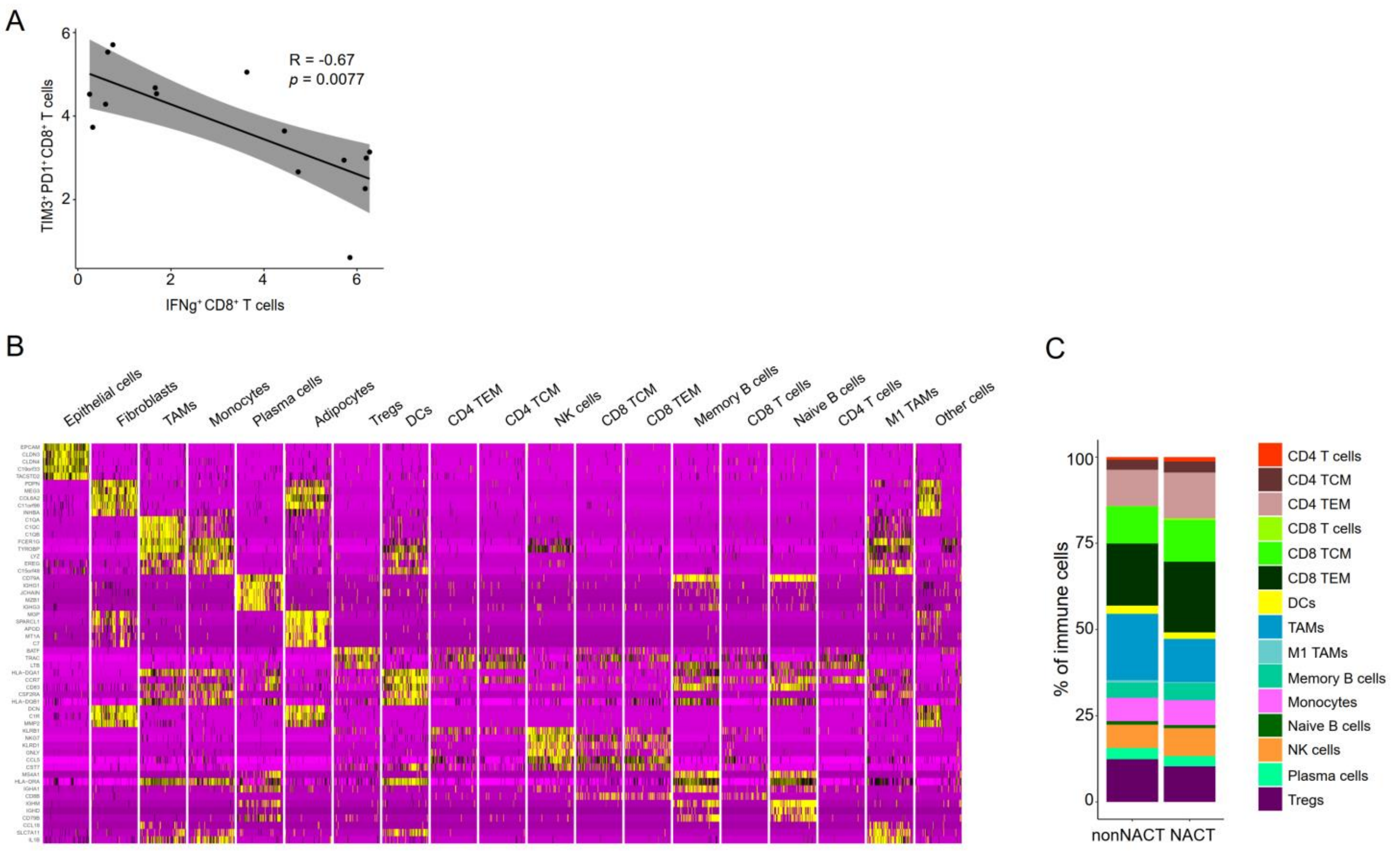

***Supplemental Figure 11*. Immunomodulation by neo-adjuvant chemotherapy in metastatic HGSOC. (A)** Correlation between frequency of TIM3$^+$PD1$^+$CD8$^+$ T cells and IFNG$^+$CD8$^+$ T cells in mTME of chemo treated HGSOC as determined by flow cytometry. *p* values are indicated. R, Pearson correlation coefficient. **(B, C)** Heatmap showing the expression of key genes representing individual cell clusters in Figure 5F, as determined by scRNAseq **(C).** The percentage of individual immune cells before and after NACT in mHGSOC, as determined by scRNAseq (study cohort 5).

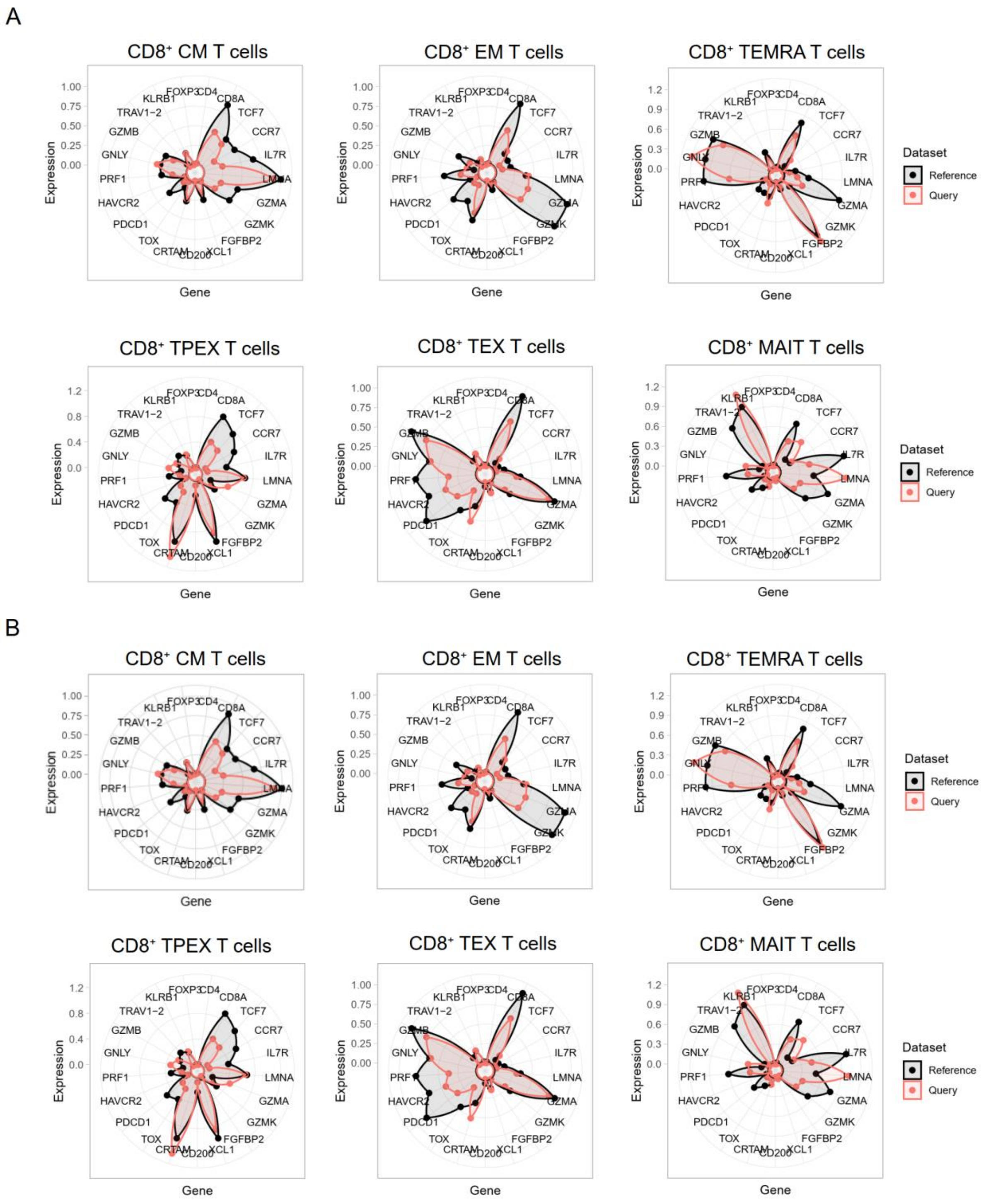

Supplemental Figure 12. **NACT mediated progenitor TCF1⁺PD1⁺CD8⁺ T cells phenotype associates with effector cytotoxic functions within metastatic HGSOC. (A, B)** Radar plot of normalized expression of T-cell gene signatures associated with central memory (CM), effector memory (EM), terminally exhausted T cells (TEMRA), progenitor exhausted T cells (TPEX), exhausted T cells (TEX) and mucosal associated invariant T cells (MAIT) within chemo-naïve (A) and chemo-treated (B) HGSOC samples from Study Cohort 5 (red) and the reference cells (black) determined by scRNAseq.

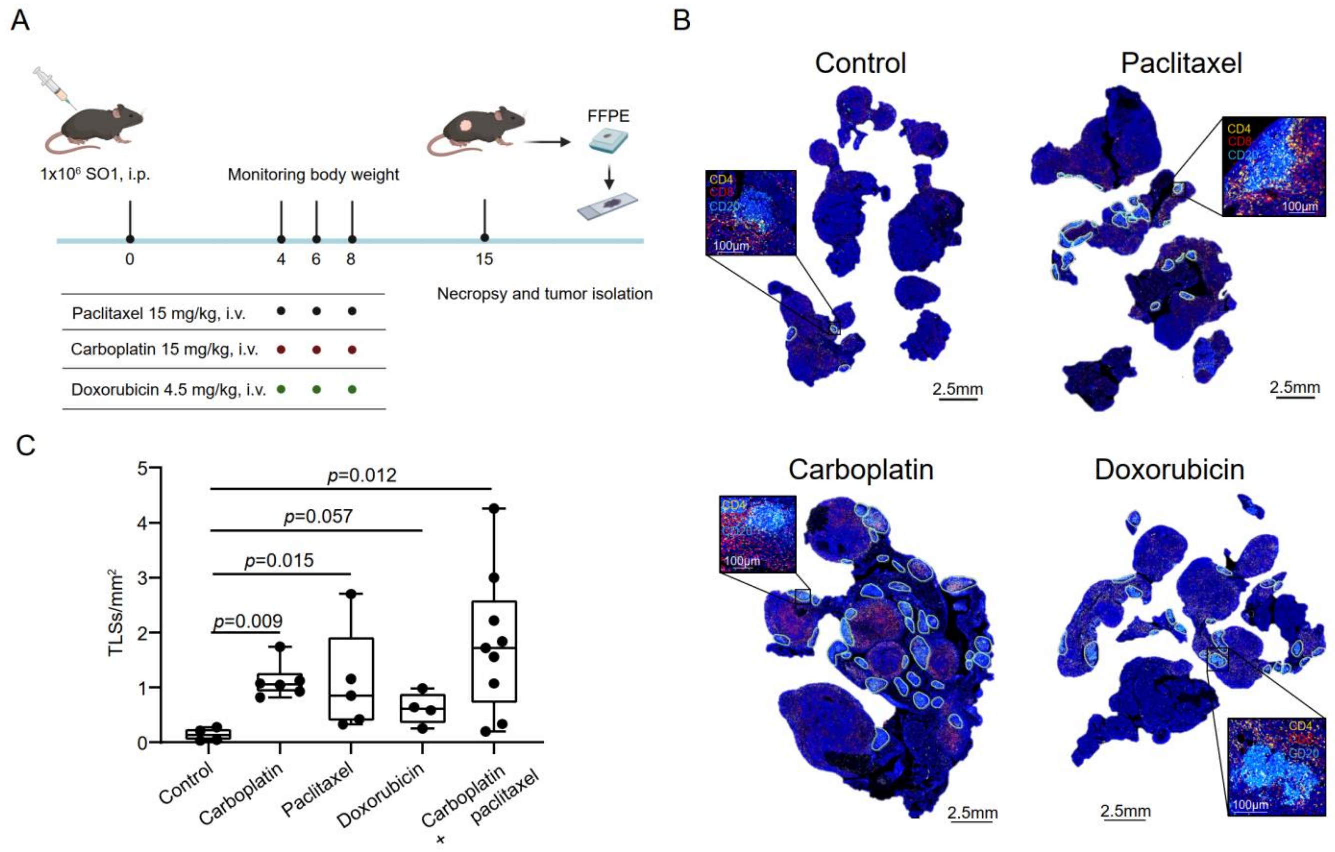

Supplemental Figure 13. **Impact of carboplatin and paclitaxel monotherapy on intratumoral TLSs development in preclinical model of ovarian cancer. (A)** Experimental design for the analysis of TLSs development after paclitaxel, carboplatin and doxorubicin monotherapy in SO1 experimental syngeneic mouse model. Created with BioRender.com. **(B, C)** Representative immunostaining for CD4, CD8 and CD20 (B) and a box plot showing density of TLSs within chemo naïve, carboplatin, paclitaxel, doxorubicin and carboplatin and paclitaxel doublet treated SO1 ovarian tumors (C). Scale bars 100µm and 2.5 mm. Box plots: lower quartile, median, upper quartile; whiskers, minimum, maximum. Statistical significance was calculated by two-sided Mann–Whitney test. *p* values are indicated.

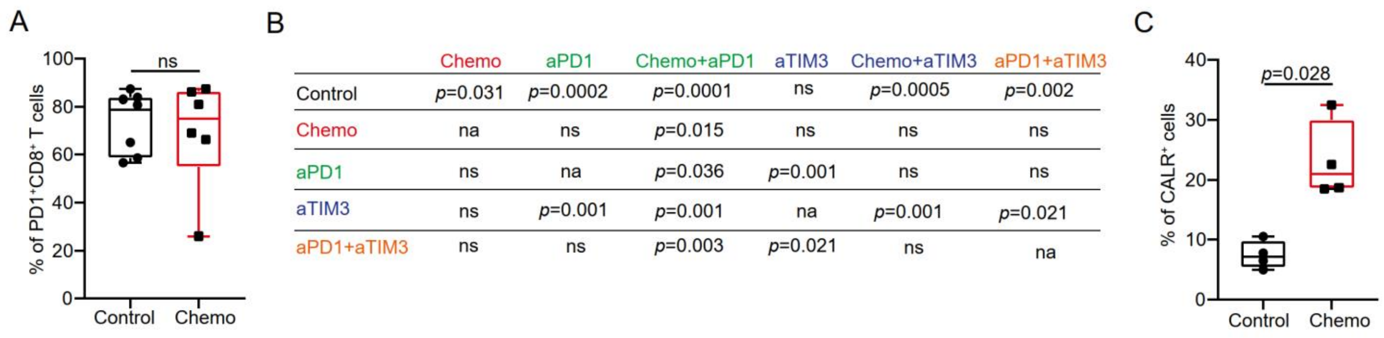

	Chemo	aPD1	Chemo+aPD1	aTIM3	Chemo+aTIM3	aPD1+aTIM3
Control	*p*=0.031	*p*=0.0002	*p*=0.0001	ns	*p*=0.0005	*p*=0.002
Chemo	na	ns	*p*=0.015	ns	ns	ns
aPD1	ns	na	*p*=0.036	*p*=0.001	ns	ns
aTIM3	ns	*p*=0.001	*p*=0.001	na	*p*=0.001	*p*=0.021
aPD1+aTIM3	ns	ns	*p*=0.003	*p*=0.021	ns	na

Supplemental Figure 14. **The clinical relevance of combinated chemo- and immuno-therapy in mouse models of TMB^{Lo} and TMB^{Hi} ovarian cancer. (A)** Flow cytometry analyses for percentages of PD1^{+}CD8^{+} T cells in tumor samples of the TMB^{Hi} SO1 experimental model in the presence or absence of carboplatin and taxane chemotherapy (chemo). Box plots: lower quartile, median, upper quartile; whiskers, minimum, maximum. Statistical significance was calculated by two-sided Mann–Whitney test. *p* values are indicated. **(B)** Overall survival (OS) of TMB^{Hi} SO1 experimental model after chemo, aPD1, aTIM3 and combined therapy (**see Fig. 6I**). Survival curves were estimated by the Kaplan–Meier method and differences between groups were evaluated using log-rank test. *p* values related to Kaplan-Meier curve in **Fig. 6I** are presented in table. **(C)** Flow cytometry analyses for percentage of CALR on ID8 cell line in absence and presence of carboplatin and taxane chemotherapy. Box plots: lower quartile, median, upper quartile; whiskers, minimum, maximum. Statistical significance was calculated by two-sided Mann–Whitney test. *p* values are indicated.

Supplemental Tables

***Supplemental Table 1.* The main clinicopathological characteristics of 110 HGSOC patients from retrospective study group.**

Variable	Cohort 1 (n=70)	Cohort 2 (n=40)
**Age**		
Mean Age	60	61
Range	36-83	30-80
**pTNM stage**		
Stage III	61 (87%)	36 (90%)
IIIA	7 (10%)	6 (15%)
IIIB	7 (10%)	4 (10%)
IIIC	47 (67%)	26 (65%)
Stage IV	9 (13%)	4 (10%)
**Debulking**		
R0	21 (30%)	17 (42.5%)
R1	6 (9%)	4 (10%)
R2	43 (61%)	19 (47.5%)
**Tumor localization**		
pTME	70	40
mTME	60	40
**Neoadjuvant chemotherapy**		
CBDCA+PTX	0 (0%)	40 (100%)
Non therapy	70 (100%)	0 (0%)
**Vital status of patients**		
Alive	16 (23%)	5 (12.5%)
Death	53 (76%)	35 (86.5%)

Abbreviations. CBDCA, carboplatin; pTME, primary tumor microenvironment; mTME, metastatic tumor microenvironment; PTX, paclitaxel

Supplemental Table 2. **The main clinicopathological characteristics of 40 HGSOC patients involved in the prospective study group.**

Variable	Cohort 3 (n=39)	Cohort 4 (n=19)
**Age**		
Mean Age	59	64
Range	38-75	36-77
**pTNM stage**		
Stage III	35 (90%)	18 (95%)
IIIA	5 (13%)	0 (0%)
IIIB	5 (13%)	3 (16%)
IIIC	24 (62.5%)	15 (79%)
Stage IV	4 (10%)	1 (5%)
**Debulking**		
R0	12 (31%)	9 (47%)
R1	6 (15%)	1 (5%)
R2	8 (21%)	5 (26%)
NA	13 (33%)	4 (21%)
**Tumor localization**		
pTME	39	19
pTME	20	19
**Neoadjuvant chemotherapy**		
CBDCA+PTX	0 (0%)	19 (100%)
3x cycles	0 (0%)	18 (95%)
4x cycles	0 (0%)	1 (5%)
Non therapy	39 (100%)	0 (0%)
**Vital status of patients**		
Alive	26 (66.7%)	13 (68%)
Death	13 (33.3%)	6 (32%)

Abbreviations. CBDCA, carboplatin; pTME, primary tumor microenvironment; mTME, metastatic tumor microenvironment; PTX, paclitaxel

Supplemental Table 3. **Antibodies and detection systems used for immunohistochemistry and immunofluorescence staining.**

Parameter	Source	Producer	Clone	Detection system	Revelation	Dilution	Incubation time [min]
**Immunohistochemistry**							
**CALR**	mouse	Abcam	FMC75	Donkey anti-mouse IgG-biot (Jackson ImmunoResearch)	DAB+ substrate Chromogen system	1:200	120
**CD4**	rabbit	LSBio	RBT-CD4	Impress HRP anti-rabbit IgG (Peroxidase) Polymer Detection kit	DAB+ substrate Chromogen system	1:50	120
**CD4**	rabbit	Abcam	EPR19514	Impress HRP anti-rabbit IgG (Peroxidase) Polymer Detection kit	AEC+ substrate Chromogen system	1:150	60
**CD8**	rabbit	Abcam	CAL38	Impress EXCEL Amplified anti-rabbit IgG kit; Impress HRP anti-goat IgG (peroxidase) Polymer Detection kit	ImmPACT DAB EqV Substrate Kit	1:250	60
**CD20**	mouse	Dako	L26	ImmPRESS-AP anti-mouse IgG (alkaline phosphatase) Polymer Detection Kit	AEC+ substrate Chromogen system	1:50	60
**CD20**	rabbit	Abcam	SP32	Impress HRP anti-rabbit IgG (Peroxidase) Polymer Detection kit	AEC+ substrate Chromogen system	1:100	60
**CD20***	mouse	Dako	L26	ImmPRESS-AP anti-mouse IgG (alkaline phosphatase) Polymer Detection Kit	ImmPACT Vector red Alkaline Phosphatase substrate kit	1:250	60
**CD21/CR2**	mouse	Cell signaling	2G9	Impress HRP anti-mouse IgG (Peroxidase) Polymer Detection kit	AEC+ substrate Chromogen system	1:50	60
**CD21**	rabbit	Abcam	EP3093	Impress HRP anti-rabbit IgG (Peroxidase) Polymer Detection kit	AEC+ substrate Chromogen system	1:500	60
**CD23**	rabbit	Abcam	SP23	Impress HRP anti-rabbit IgG (Peroxidase) Polymer Detection kit	AEC+ substrate Chromogen system	1:200	60
**CD57**	mouse	Abcam	NK/804	Impress HRP anti-mouse IgG (Peroxidase) Polymer Detection kit	AEC substrate system	1:50	120
**CXCR5**	rabbit	Abcam	EPR23463-30	Impress HRP anti-rabbit IgG (Peroxidase) Polymer Detection kit	AEC substrate system	1:500	60
**DC-LAMP***	rat	Dendritics	1010E1.01	Donkey anti-rat IgG-biot (Jackson ImmunoResearch)	DAB+ substrate Chromogen system	1:80	60
**FoxP3**	mouse	Abcam	236A/E7	Impress HRP anti-mouse IgG (Peroxidase) Polymer Detection kit	AEC substrate system	1:50	120
**PD1**	rabbit	Abcam	CAL20	poly-HRP-conjugated sec.Ab goat anti-rabbit + Biotin XX Tyramide reagent + Streptavidin-HRP	AEC substrate system	1:50	120
**PanCK**	mouse	Abcam	AE1/AE3	Impress HRP anti-mouse IgG (Peroxidase) Polymer Detection kit	AEC substrate system	1:150	60
**TCF1/TCF7**	rabbit	Cell signaling	C63D9	Impress HRP anti-rabbit IgG (Peroxidase) Polymer Detection kit	AEC substrate systém	1:50	120

**TIM3**	rabbit	Cell signaling	D5D5R	poly-HRP-conjugated sec.Ab goat anti-rabbit + Biotin XX Tyramide reagent + Streptavidin-HRP	AEC substrate system	1:50	120

***CD20/DC-LAMP was stained in double staining protokol**

Immunofluorescence

**CD8**	rabbit	Abcam	SP16	poly-HRP-conjugated sec.Ab goat anti-rabbit	Alexa Fluor™ 594 Tyramide reagent	1:60	90
**CD20**	mouse	Dako	L26	goat anti-mouse IgG (H+L cross absorbed secondary antobody A750	NA	1:300	60
**CD68**	mouse	Abcam	KP1	poly-HRP-conjugated sec.Ab goat anti-mouse	Alexa FluorTM 647 Tyramide Reagent	1:30	overnight
**CD163**	rabbit	Abcam	EPR19518	poly-HRP-conjugated sec. Ab goat anti-rabbit	Alexa FluorTM 488 Tyramide Reagent	1:125	90
**DC-LAMP**	rat	Dendritics	1010E1.01	Immpress -HRP anti-rat IgG polymer	Alexa Fluor™ 546 Tyramide reagent	1:350	overnight
**GZMB**	rabbit	Abcam	EPR8260	poly-HRP-conjugated sec.Ab goat anti-rabbit	Alexa Fluor™ 488 Tyramide reagent	1:250	60

Ultivue

**CD8**	na	Ultivue	C8/144B	FITC	Ultivue (manufacturer´s instructions)
**PD1**	na	Ultivue	CAL 20	TRIC	Ultivue (manufacturer´s instructions)
**PD-L1**	na	Ultivue	73-10	Cy5	Ultivue (manufacturer´s instructions)
**CD68**	na	Ultivue	KP-1	Cy7	Ultivue (manufacturer´s instructions)
**GZMB**	na	Ultivue	EPR8260	FITC	Ultivue (manufacturer´s instructions)
**CD4**	na	Ultivue	SP35	TRIC	Ultivue (manufacturer´s instructions)
**FoxP3**	na	Ultivue	236A/E7	Cy5	Ultivue (manufacturer´s instructions)
**CD20**	na	Ultivue	L26	Cy7	Ultivue (manufacturer´s instructions)

***Supplemental Table 4*. The list of antibodies used for flow cytometry.**

Parameter	Source	Producer	Clone	Fluorochrome	Dilution
**CD3**	mouse	Exbio	MEM-57	A700	5:100
**CD8**	mouse	BD Biosciences	RPA-T8	HV 500	5:100
**CD45**	mouse	Exbio	MEM-28	PerCP	6:100
**CD45**	mouse	Thermo Fisher Scientific	HI30	PE-Texas Red	6:100
**PD1**	mouse	BioLegend	EH12.2H7	FITC	6:100
**TIM3**	mouse	BioLegend	F38-2E2	PE	3:100
**GZMB**	mouse	BD Biosciences	GB11	BV421	4:100
**CD45**	rat	Invitrogen	30-F11	eF506	0.5:100
**CD3**	rat	BioLegend	17A2	PerCP Cy 5.5	1:100
**CD8a**	rat	BD BioSciences	53-6.7	BV605	1:100
**PD1**	rat	BioLegend	RMP1-23	APC	1:100
**TIM3**	rat	BD BioSciences	RMT3-23	BV421	4:100
**TCF1**	rabbit	Cell Signaling	C63D9	NA	0.25:100
**Anti-rabbit IgG**	goat	Cell Signaling	NA	PE	1:600
**CALR**	mouse	Enzo Life eciences, Ing	FMC75	-	2,4:100
**Epitelial antigen**	mouse	DAKO	Ber-EP4	FITC	2:100
**Epcam**	mouse	BioLegend	9C4	FITC	2:100
**Pan Cytokeratin**	murine	eBioscience	AE1/AE3	A488	2:100
**CD62L**	rat	BD BioSciences	MEL-14	FITC	1:100
**CD44**	rat	BD BioSciences	IM7	PE-CF594	1:100

Supplemental Table 5. Gene expression signature associated with CD8$^+$ T cells, B cells, cytotoxicity, myeloid dendritic cells (mDCs), tertiary lymphoid structures (TLS) and immunosuppression as determined on RNAseq data from pTME and mTME HGSOC with/without NACT (heatmap presented in Fig. 1D). *p* values are presented.

	pTME		mTME	
	**p.value**	**Hodges-Lehmann**	**p.value**	**Hodges-Lehmann**
**T cells**	0.0029	0.44	0.0186	0.41
**CD8 T cells**	0.0000	1.33	0.0013	1.33
**Cytotoxic T cells**	0.0002	0.54	0.0015	0.75
**B cells**	0.0003	0.81	0.0472	0.63
**NK cells**	0.0890	0.15	0.0020	0.30
**Monocytes**	0.0036	0.53	0.0013	0.64
**mDCs**	0.0003	0.36	0.0989	0.31
**Immunosuppression**	0.0106	0.46	0.0004	0.86
**T cell activation**	0.0710	0.45	0.3611	0.21
**T cell survival**	0.0287	0.32	0.0538	0.40
**TREGs**	0.4932	0.11	0.4855	0.15
**MHCI**	0.4429	0.22	0.2257	0.42
**Myeloid cell chemotaxis**	0.0560	0.50	0.0126	0.85
**TLS signature**	0.0029	0.64	0.0201	0.55
**PD1**	0.1340	0.28	0.0574	0.42
**PDL1**	0.0243	0.39	0.2821	0.28
**PDL2**	0.0010	0.79	0.0252	0.53
**CTLA4**	0.0215	0.46	0.0989	0.55
**TIM3**	0.0287	0.47	0.0076	0.55
**LAG3**	0.0183	0.62	0.0933	0.45
**TIGIT**	0.0145	0.40	0.0413	0.50

Abbreviations: mDCs, myeloid dendritic cells; NK cells, natural killer cells; TREGs, regulatory T cells; TLS, tertiary lymphoid structures
